# Structural and Phylogenetic Analysis of SARS-CoV-2 Spike Glycoprotein from the Most Widespread Variants

**DOI:** 10.3390/life12081245

**Published:** 2022-08-16

**Authors:** Emilia Caputo, Luigi Mandrich

**Affiliations:** 1 Institute of Genetics and Biophysics-IGB-CNR, “A. Buzzati-Traverso”, Via Pietro Castellino 111, 80131 Naples, Italy; 2 Research Institute on Terrestrial Ecosystems-IRET-CNR, Via Pietro Castellino 111, 80131 Naples, Italy

**Keywords:** SARS-CoV-2, SARS-CoV-2 spike protein, SARS-CoV-2 variants, structural analysis

## Abstract

The SARS-CoV-2 pandemic, reported for the first time at the end of 2019 in the city of Wuhan (China), has spread worldwide in three years; it lead to the infection of more than 500 million people and about six million dead. SARS-CoV-2 has proved to be very dangerous for human health. Therefore, several efforts have been made in studying this virus. In a short time, about one year, the mechanisms of SARS-CoV-2 infection and duplication and its physiological effect on human have been pointed out. Moreover, different vaccines against it have been developed and commercialized. To date, more than 11 billion doses have been inoculated all over the world. Since the beginning of the pandemic, SARS-CoV-2 has evolved; it has done so by accumulating mutations in the genome, generating new virus versions showing different characteristics, and which have replaced the pre-existing variants. In general, it has been observed that the new variants show an increased infectivity and cause milder symptoms. The latest isolated Omicron variants contain more than 50 mutations in the whole genome and show an infectivity 10-folds higher compared to the wild-type strain. Here, we analyse the SARS-CoV-2 variants from a phylogenetic point of view and hypothesize a future scenario for SARS-CoV-2, by following its evolution to date.

## 1. Introduction

### 1.1. General Information

SARS-CoV-2 is a severe infection reported at Wuhan city in China at the end of 2019, caused by a new type of coronavirus. It has spread across several countries in the world with a very high speed, causing several dead, mainly because it was unknown. Moreover, the intense trade and tourism exchanges from and to China has been another critical factor affecting its fast spreading. Thus, in February 2020, the World Health Organization (WHO) declared the pandemic state.

In March 2021, after one year of the pandemic, WHO published in a report that more than 150 million people have been infected, and about three million have died from COVID-19 (Coronavirus Disease-19 due to the SARS-CoV-2 virus; https://www.who.int/pubblications, accessed on 30 March 2021).

In April 2022, WHO confirmed globally over 514 million of infection cases and over six million deaths (https://www.who.int/publications/m/item/weekly-epidemiological-update-on-covid-19, accessed on 27 April 2022).

The response to the pandemic was the development of vaccines in less than one year; in fact, starting from December 2020, five vaccines have been licensed and used [1,2]. To date, over 11 billion doses of vaccines have been consumed; in some European countries, the immunized people percentage value reached about 80%. From the beginning of the pandemic, more than 100 different vaccines have been developed; moreover, about 30 of these have been evaluated in phase III of clinical trials [3]. In the case of SARS-CoV-2, beyond traditional viral vector vaccines, inactivated vaccines and protein-based vaccines, and for the first time, mRNA vaccines, have been developed and used [3]. Despite these excellent results, SARS-CoV-2 is still considered very dangerous. In fact, from June to July 2022, the weekly cases reported by WHO, have been about six million; while the weekly deaths have been about 11,000. For this reason, large areas have been in lockdown to limit the virus spreading in some countries, such as North Korea (in March 2022) and a restricted zone in Wuhan, China (July 2022) (https://www.who.int/docs/default-source/coronaviruse/situation-reports/20220720_weekly_epi_update_101.pdf?sfvrsn=7fbbc4ef_3&download=true, accessed on 20 July 2022).

From a symptomatic point of view, the infection needed approximately four days of incubation, and it is typically characterized by a dry cough and fever. In most patients, COVID-19 infection remains mild or with moderate symptoms that are solved within a week. At the beginning of the pandemic, about 10% of patients showed symptoms through the second week of infection; these are associated with a high risk of developing a more severe COVID-19 disease. The most common and severe symptoms observed during the infection were: respiratory (cough, sputum, and conjunctivitis); musculoskeletal (headache, fatigue, and myalgia); gastrointestinal (abdominal pain, diarrhea, vomiting, and intestinal inflammation); cutaneous (erythema multiform and a chickenpox-like rash); and nervous systems (anosmia, dysgeusia, myalgia, and the loss of speech or movement) [4].

Several studies have been conducted in order to elucidate the SARS-CoV-2 mechanism of action. The critical role of the spike glycoprotein (S) [5,6] has been highlighted; furthermore, interestingly, it has been observed that SARS-CoV-2 is characterized by a high number of mutations in its genome [7].

In these two years, various SARS-CoV-2 variants have been sequenced. They are characterized by mutations in the spike protein. Therefore, since the vaccines have been developed using the spike protein as an antigen, they have showed a reduced efficacy against the last variants due to the accumulation of mutations on the spike protein [8].

Here, we analyzed, at a structural and phylogenetic level, the spike proteins characterizing the SARS-CoV-2 variants most diffused and sequenced in the last two years.

### 1.2. SARS-CoV-2 Virus

SARS-CoV-2 belongs to the *Coronaviridae* family [9]; its genome is a positive sense single-stranded RNA of about 30 kb length; it encodes: 16 non-structural proteins, namely nsp 1–16; the ORFs accessory proteins 3a, 6, 7a, 7b, 8, and 10; and four structural proteins involved in the viral infection, which are the spike protein (S), membrane protein (M), nucleocapsid protein (N), and envelope protein (E) [10,11,12]. The SARS-CoV-2 genome is not segmented RNA, having the 3′ poly-A tail and 5′ cap structure.

**Figure 1:**
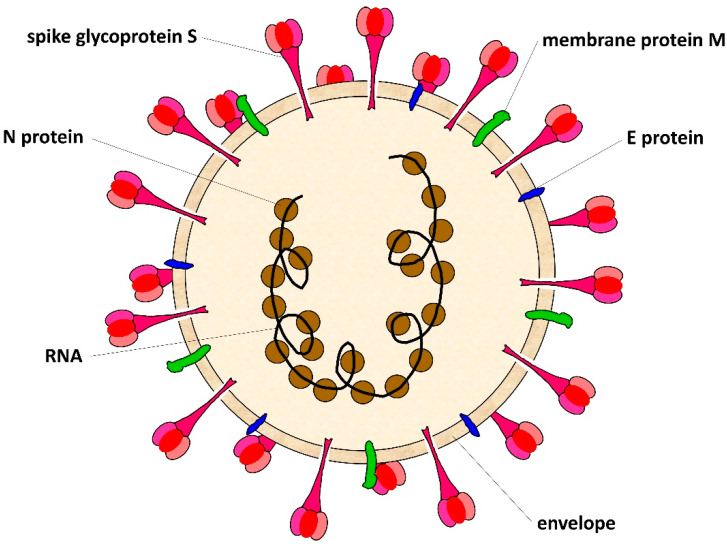
**SARS-CoV-2 virus section**. SARS-CoV-2 is an enveloped virus with a spherical morphology and a diameter of 100–150 nm. The lipidic envelope derives from the host cell. On the viral surface are present different types of protein, such as the E protein, M membrane protein, and spike protein. The viral ssRNA is surrounded by the N nucleoproteins.

The SARS-CoV-2 shows a spherical morphology with a diameter of about 100–150 nm [13]. A schematic representation of SARS-CoV-2 is reported in Figure 1: the ssRNA, which is surrounded by the N proteins, is inside of the lipidic envelope; and associated to the membrane there are the glycoproteins M, the proteins E, and the spike glycoprotein that protrudes from the surface of the virus in a high number of copies to look like a crown.

During the infection, SARS-CoV-2 uses the spike protein for binding to the host cell receptor: the ACE-2 protein (angiotensin-converting enzyme 2). The process takes place in several steps, which require, at first, the spike protein cleavage for its interaction with the ACE-2 protein; then, there is the fusion of the cell-virus membranes [14,15]; this is followed by the entry into the host cell of viral ssRNA [14,15]. Since the spike protein is crucial for the virus infection and it is present on the virus surface in a high number of copies, vaccines have been produced by using this protein as a target [16].

### 1.3. SARS-CoV-2 Mutation Rate

Single-strand RNA viruses show an increased mutation rate compared to the ones having DNA genomes, since they lack the systems to correct the replication errors [17]. Further, these viruses exhibit a consistent mutation-rate variability among them; this ranges between 10^−6^ and 10^−3^, where the mutation rate is defined as nucleotide (nt) substitutions per-site per-cell infection [18]. It is not easy to measure the real mutation rate of a virus, because most of the mutations are lethal for the virus.

The mutation frequency of SARS-CoV-2 depends on the probability that an error occurs during the genome replication, and it has been calculated as three in a million. Therefore, three replications per million are different from the parental strain; whereas the mutation rate of SARS-CoV-2 *per-site per-year* has been estimated as 1.12 × 10^−3^ nt^−1^ year^−1^ [19,20].

Moreover, fixed mutations have been identified in the genome by the whole viral genome analysis; this suggests that SARS-CoV-2 changed much more slowly than other ssRNA viruses [21].

However, taking into consideration its spread throughout the world and the number of viral replications in a single infected person, all together these data may explain the observed accumulation of thousands of mutations. Furthermore, from additional data about this aspect, it has been determined that individuals with a high viral load may generate up to 1.23 × 10^5^ copies of viral RNA from a single cough; however, individuals with a moderate viral load can generate only a few hundred copies [22].

Since the spike protein is one of the most important viral proteins involved in the infection mechanisms, it has been used as the main antigen to produce vaccines to fight the SARS-CoV-2 pandemic [1,23]. It has also been used in diagnostics to classify and to monitor all the viral variants derived from the mutations sequenced on the spike protein; these are known as the variants of concern (VOC) [23].

### 1.4. Spike Protein S

**Figure 2:**
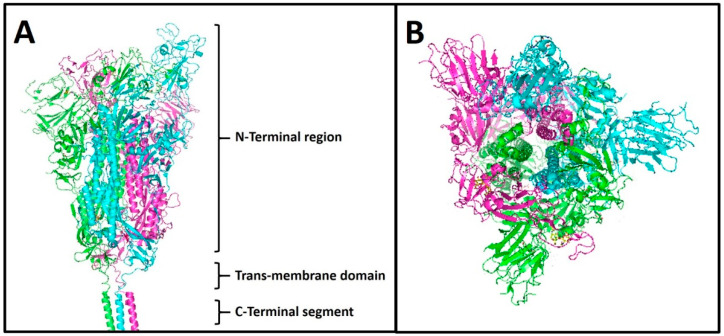
**3D structure of spike protein**. The 3D structure was obtained by cryo-electron microscopy at a resolution of 3.5 Å, by Wrapp and colleagues (Science, 2020), PDB ID: 6VSB. (**A**) The whole visualization of the 3D homo-trimer structure of the spike protein. The monomers are indicated with different colours: magenta, green, and cyan. (**B**) The top view of the spike homo-trimer.

The SARS-CoV-2 spike protein is a glycoprotein consisting of 1273 amino acids, with a molecular weight of about 180–200 kDa. It is localised on the virus surface in a high number of copies as a pre-fusion protein (Figure 1), with a homo-trimer conformation where each monomer lacks the signal peptide (amino acids 1–13). The 3D structure has been solved by cryo-electron microscopy at a resolution of 3.5 Å (Figure 2); functionally, the spike can be divided into an extracellular N-terminal region, a trans-membrane domain (23 residues), and a short intracellular C-terminal segment (39 residues) (Figure 2A) [24]. Morphologically, the N-terminal region of spike is very large compared to the other protein regions, forming a characteristic bulbous (Figure 2B); in addition, it undergoes an extensive structural rearrangement when it interacts with the receptor of the host cell.

**Figure 3:**
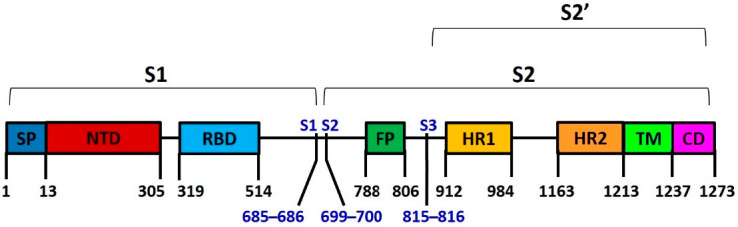
**Schematic representation of spike protein**. The functional domains are indicated as subunit S1 (residues 14–685) and subunit S2 (residues 686–1273) responsible for receptor binding and membrane fusion, respectively. S1, S2, and S3 are the cleavage sites at positions 685, 699, and 815, respectively. The cleavage at position 815 generates the subunit S2′. SP is the signal peptide (residues 1–13); NTD is the N-Terminal domain (residues 14–305); RBD is the receptor-binding domain (residues 319–514); FP is the fusion peptide (residues 788–806); HR1 is the heptapeptide repeat sequence 1 (residues 912–984); HR2 is the heptapeptide repeat sequence 2 (residues 1163–1213); TM is the transmembrane domain (residues 1214–1237); CD is the cytoplasmatic domain (residues 1238–1273).

In detail, Figure 3 schematically represented the primary structure of the spike protein: at the N-terminal, the residues 1–13 are the signal peptide, which is removed during membrane migration; the residues 14–685 are indicated as subunit S1; and the residues 686–1273 are indicated as subunit S2. Between these two subunits, there are, at positions 685 and 699, two cleavage sites that are necessary to cut the spike. These cleavages, as mentioned above, are critical to activate the membrane fusion process with the host cell [24].

The S1 subunit is responsible for the receptor binding to the host cell by the recognition of the angiotensin converting enzyme 2 (ACE-2), while the S2 subunit is responsible for the membrane fusion; each subunit is divided into different subdomains, as indicated in Figure 3. The receptor-binding domain (RBD) changes its position during the interaction with ACE-2. In particular, the spike protein opens its conformation by moving RBD versus ACE-2 (open conformation) [25]. Then, after membrane fusion, the subunit S2 is cut at position 815; this originates the S2′ subunit (Figure 3) [24,25].

**Figure 4:**
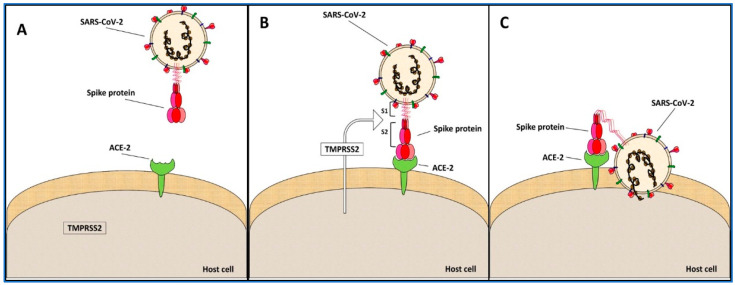
**SARS-CoV-2 infection mechanism**. (**A**) *Initial state*: it is represented by the separated virus and host cell. (**B**) *Binding*: the spike protein recognizes ACE-2 and starts the binding process that involves the TMPRSS2 serine protease from the host cell; the protease cuts the spike protein between the subunits S1 and S2. (**C**) *Cell fusion*: the cleavage activates the spike protein, which changes its conformation, bending towards the cell membrane to which it merges; then, the genetic material of the virus enters into the host cell, starting the viral duplication.

The spike protein is localized on the virus surface, in a non-active form (closed conformation) (Figure 4A). Following the infection process, the RBD domain binds ACE-2 (open conformation); the transmembrane serine protease 2 (TMPRSS2) from the host cell recognizes and cuts the spike into the S1 and S2 subunits (Figure 4B). Then, by this event, the membrane fusion process starts. In particular, the hydrophobic FP domain anchors the spike to the host membrane; the domains HR1 and HR2 of the three monomers form a six-helical bundle, bringing the viral envelope to the host cell membrane and completing the fusion process (Figure 4C). The last part of infection is the injection of the viral ssRNA into the host cell, leading to the replicative viral process [26].

### 1.5. Spike Variants and Phylogenies

Casual mutations occur during the viral RNA replication, generating new SARS-CoV-2 variants. Only some of these mutations had an effect on the processes of viral infection and diffusion, as well as on the virus-induced symptoms; others are harmful for the survival of the virus and are eliminated, and some are neutral and are accumulated in the genome. In the three years of the pandemic, many SARS-CoV-2 variants have been observed and sequenced [27]. The spike protein has been used as the primary antigen for the vaccine production because it is crucial for the infection mechanism and it is a characterising SARS-CoV-2 protein. Further, the mutations observed in the gene coding this protein have been used for the classification of the viral variants [28]. The most diffused SARS-CoV-2 variants have mutations capable of changing the virus infection and diffusion processes; they have spread, replacing the wild-type or the previous virus variants [29,30].

The most recent SARS-CoV-2 variants have accumulated many mutations on the spike protein. Thus, since the vaccines have been developed on the wild-type version of spike, many cases of reinfection by COVID-19 and infections in vaccinated people have been observed [31].

Here, we analysed the most diffused SARS-CoV-2 variants by sequence alignment, in relation to the timing of their spreading and the place where they were first isolated. We did so in order to perform a phylogenetic analysis to understand the genetic evolution of the virus, and to define the characterizing mutations of SARS-CoV-2 (Table 1) during its evolution.

The twenty most diffused SARS-CoV-2 spike variants, identified from December 2019 to the beginning of 2022, indicated as wild type, delta, lambda, mu, beta, gamma, B.1.1.318, kappa, A 23.1, iota, theta, epsilon, 20 A.EU1, 20 A.EU2, zeta, alpha, eta, omicron BA.1, omicron BA.2, omicron BA.2.12.1, omicron BA.4, and omicron BA.5 (sequences were from proteins database at https://www.uniprot.org, accessed on 1 December 2003) were selected for the analysis. The sequences of these variants were used to make the multiple sequence alignment reported in Figure S1 (see supplemental data) (the multiple sequence alignment by the Clustal Omega program at https://www.ebi.ac.uk/Tools/msa/clustalo/, accessed on 1 October 2019). From the alignment is derived the phylogenetic tree of the variants that will be discussed below.

We found 100 mutations in total, including residue substitutions, deletions, and insertions, which are about 8% of the total residues (1273 aa). Moreover, 49 of these mutations were included in NTD and 23 in the RBD domain; they were comprised in more than 70% of the S1 spike subunit mutations (Figure 3).

These data suggested that the greatest changes occur in the recognition region of the ACE-2 host cell receptor [32], while the spike protein domains involved in the conformational changes and in its activation did not vary. Further, these results supported that the virus infection rate variation observed in these variants was correlated to the change of the binding affinity of the spike protein to the ACE-2 protein [32].

Interestingly, only the residue D614 is mutated in all the examined variants, excluding the variant A 23.1, that maintain D614 (Figure S1, supplemental data); this observation indicates that one of the first mutations that occurred in the spike was at position 614, and it is known that the mutation of this residue impacts on the functionality of the protein [33].

Among the other mutations, the most diffused are at the following positions: 452 (in 7 variants); 484 (in 13 variants); 501 (in 10 variants); and 681 (in 12 variants). Further, 51 mutations are unique for the variants; among them, 20 have been identified only in the Omicron variants (Table 2).

Furthermore, we observed that the “unique” mutations listed in Table 2 cannot be considered variant-specific and all characterising the SARS-CoV-2 variants; this is because in most cases they are substitutions between amino acids with similar properties, not affecting the spike protein functionality. For instance, in the 20 A.EU 1 and 2 variants, the spike protein differs from the wild type by the same single residue (D614G) in both. In addition, another two mutations were found: the S477N in the variant 20 A.EU 2; and the A222V in the variant 20 A.EU 1. The mutation S477N was also identified in other spike variants, while A222V was observed only in 20 A.EU 1; this suggests that the functional difference between the two 20 A.EU variants is due to the S477N mutation and not due to the A222V, being a conservative substitution of a non-polar amino acid with a similar one [34]. Based on these observations, if the mutation S477N is characterising between these two variants and it is also present in other more recent variants, 20 A.EU 2 may be considered an old variant, where the mutation S477N appeared for the first time.

In the case of the variant named Iota, some mutations are common to other variants, such as S477N, E484K, and D614G; however, the two mutations found only in Iota, L5F, and D253G can be considered characterizing because they have an effect on the functionality of the spike. In fact, the L5F mutation is localized in the signal peptide (SP) of the spike; it has been observed that mutations of SP alter the spike functionality [35]. The second mutation D253G is the substitution of polar residues (aspartic, D) with non-polar ones (glycine, G), generating a consistent change.

Other characterising mutations observed in the spike variants affecting its functionality are: the deletion 156–158 in Delta; the deletion 241–243 in Beta; and the deletion 246–252 in Lambda (Table 2).

### 1.6. The Omicron Variants

At the end of 2021, a new SARS-CoV-2 variant named Omicron was isolated in South Africa and Botswana; it was, successively, sequenced (Organization WH. Classification of Omicron (B.1.1.529): SARS-CoV-2 variant of concern. 2021. https://www.who.intnewsitem/26-11-2021-classification-of-omicron-(b.1.1.529)-sars-cov-2-variant-of-concern, accessed on 26 November 2021).

Omicron, compared to the previously variants, is characterised by having many mutations; it has about 50 on the whole genome and 32 of them are only in the spike protein [36]. As regards its main features, Omicron shows an increased infectivity (10-folds higher with respect to the wild-type strain) and milder symptoms with respect to the original virus [36]. This variant is also able to escape the immune system of the host due to the high number of mutations on the spike protein. This led to an extremely fast spreading of the Omicron variant in South Africa, such that it completely replaced the Delta variant in only two weeks [36]. Due to its rapid spreading and high capability to mutate, from November 2021 to January 2022, four Omicron sub-variants were isolated (Table 1).

Among the mutations identified in the Omicron variants, 14 are exclusive and found in all the Omicron variants. They are: G339D, S373P, K417N, N440K, S477N, T478K, E484A, Y505H, H655Y, N679K, N764K, D796Y, Q954H, and N969K (Figure S1, supplemental data). However, six other mutations have been observed in all the Omicron variants, excluding Omicron BA.1; they are: Del24-26, V213G, T376A, S371F, D405N, and R408S (Figure S1, supplemental data).

**Figure 5:**
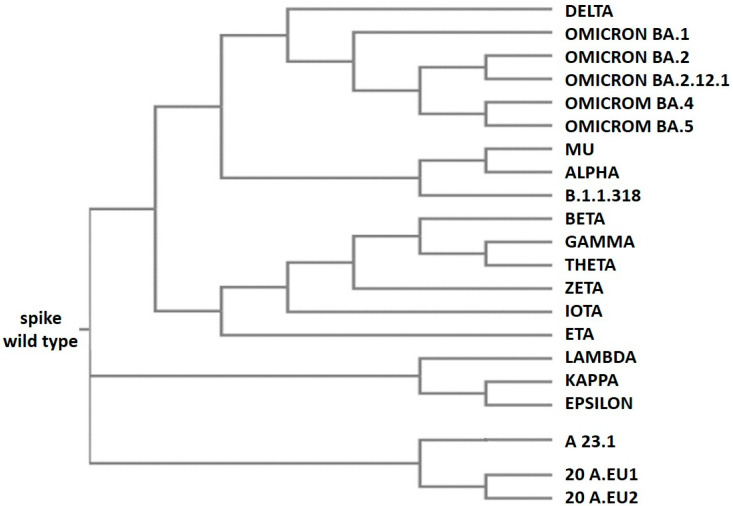
**Phylogenetic tree of SARS-CoV-2 variants**. The tree was generated by the alignment obtained using the Clustal Omega program (https://www.ebi.ac.uk/Tools/msa/clustalo/, accessed on 1 October 2019). For the alignment, the default parameters were used (dealign input sequences: no (false); number of combined iterations: 0; max guide tree iterations: −1 (off); max HMM iterations: −1 (off); use mBed-like clustering during subsequent iterations: yes (true); mBed-like clustering guide-tree: yes (true)).

From an evolutionary point of view, the Omicron variants are derived from a common “ancestral” version of the SARS-CoV-2 Alpha variant (Figure 5). In fact, they have some critical mutations in common; these are Del69-70, N501Y, D614G, and P681H, all of which are involved in the spike protein functionality [37,38,39]. Omicron BA.1 is quite different from the other omicron variants because it has 11 unique mutations with respect to the others (Table 2). For this reason, it is possible that it evolved separately from the other Omicron variants and was present at least 3–4 months before its isolation; otherwise, it could not have been so different from the other Omicron variants. These findings are evident in Figure 5, which represents the phylogenetic tree generated by the alignment reported in Figure S1 (supplemental data). In the upper part of Figure 5 are located the Omicron variants; together with Delta, they evolved from a common ancestor of the Alpha variant; and Omicron BA.1 appears in an evolutionary branch that separated early from the other Omicrons.

In the lower part of Figure 5, the SAR-CoV-2 variants grouped as A 23.1, A 20.EU 1, A 20.EU 2, and Lambda, Kappa, and Epsilon appear in others’ early evolutionary branches evolved from the initial virus. It is important to note that the variant Epsilon was isolated and sequenced in the USA in March 2020. However, the variants A 23.1 and Lambda were isolated only in October 2020 and December 2020, respectively, in Uganda and Peru; in these two countries, for technical and economic reasons, not many samples were sequenced compared to the USA. It is interesting that the two variants Delta and Kappa were isolated in India, both in December 2020 (Table 1). It is evident that their evolution is divergent, by looking at the phylogenetic tree (Figure 5). In fact, both variants carry seven mutations with respect to the wild-type protein; however, only three of them are common, specifically: L452R, D614G, and P681R. This may suggest the presence of an unknown variant intermediate carrying these three mutations, which then diverged into the two variants Kappa and Delta.

The phylogenetic tree gives a clear idea of the variants’ evolution, even if it lacks an indication of some variants being less widespread and no detected intermediate variants; this is mainly regarding the Omicron variants that have many more mutations with respect to the others. Their evolution originated through a series of intermediate variants that are currently unknown.

## 2. Conclusions

It is important to note that three years after the beginning of the SARS-CoV-2 pandemic and despite the availability of several vaccines for two years, the emergency and the restrictive measures to contain SARS-CoV-2 spreading persist in many countries. In particular, the last variants are generating new outbreaks of infection, even in countries where the level of vaccinations is very high. This is the result of various factors: the genetic evolution of the virus; the reduced effectiveness of the vaccines; and the screening systems currently used. The main problem is the continuous evolution of the virus, especially in the poorest countries where the level of vaccinations is very low and the infection rates are high. Actually, these areas are restricted in Africa, and represent the incubator for the genetic evolution of SARS-CoV-2; in fact, the Omicron variants are from South Africa.

The reduced effectiveness of the vaccines is related to the genetic evolution of SARS-CoV-2. In fact, the vaccines are less effective because they have been produced against the wild-type version of the spike protein [3]. Actually, the spike protein of the most diffused Omicron variants presents over 30 mutations compared to the wild-type version; among these, there are three deletions in the subunit S1 (Figure S1, Supplemental data), which is responsible for the receptor binding to the host cell and is considered an important target of the immune response.

For instance, in Italy, over 85% of the population received one dose of the vaccine and over 65%, two doses. However, in June 2022, about 80,000 new infected people per day were recorded; although most of them are vaccinated, an increase of reinfection in people was reported that represents about 8% of the total infections (data from the Italian Government bulletin; https://www.epicentro.iss.it/coronavirus/bollettino/Bollettino-sorveglianza-integrata-COVID-19_22-giugno-2022.pdf, accessed on 22 June 2022). Despite the high number of infections compared to the data referred to in 2020, the mortality, as well as the number of people needing hospital care, is low [40].

Another problem concerns the tests used for SARS-CoV-2 detection, since the antigenic rapid tests recognize the wild-type version of the spike protein; by using this diagnostic system against the new variants, this gives a large number of false negatives and keeps the spread of the virus at a high rate. Therefore, to reduce it, rapid tests should be developed to detect the Omicron variants.

What could be the most probable scenario to define the evolution and the end of the SARS-CoV-2 pandemic? From an evolutionary and survival point of view, we are observing the selection of variants that are more diffusible; however, at the same time, they are showing less severe symptoms in infected patients with respect to the initial virus. In fact, the symptoms that were observed in patients at the beginning of the pandemic, such as the lack of flavours, odours, erythema, and severe respiratory symptoms [4], have disappeared in the more recent Omicron variants [41,42].

The rapid spread of new variants and their symptomatic similarity to a common cold, offers indications as to how the virus is adapting and how the infection may evolve in the next months, moving from pandemic to endemic virosis; we note that the other known four human seasonal coronaviruses, HCoV-NL63, HCoV-229E, HCoV-OC43, and HCoV-HKU1 [43], which cause mild symptoms, have been circulating in humans for decades.

## Figures and Tables

**Table 1:** SARS-CoV-2 variants. The most spread SARS-CoV-2 variants, the country where they were first isolated, and the isolation dates were reported.

Spike Variants	Isolation Country	Isolation Date
wild type	China	December 2019
EPSILON	USA	March 2020
ZETA	Brazil	April 2020
BETA	South Africa	May 2020
20 A.EU2	Portugal	June2020
20 A.EU1	Spain	July 2020
ALPHA	England	September 2020
DELTA	India	October 2020
KAPPA	India	October 2020
A 23.1	Uganda	October 2020
GAMMA	Brazil	November 2020
IOTA	USA	November 2020
ETA	Multiple countries	November 2020
LAMBDA	Peru	December 2020
THETA	Philippines	January 2021
B.1.1.318	Multiple countries	January 2021
MU	Columbia	January 2021
OMICRON BA.1	South Africa	November 2021
OMICRON BA.2	South Africa	December 2021
OMICRON BA.2.12.1	North America	December 2021
OMICRON BA.4	South Africa	January 2022
OMICRON BA.5	South Africa	January 2022

**Table 2:** A list of single mutations in the spike variants. For each variant, only the exclusive and unique mutations sequenced were reported. “Del” is a deletion of residues; “Ins” is an insertion of residues.

Spike Variants	Mutations
ALPHA	A570D T716I S982A D1118H
BETA	D80A D215G Del241-243 K417N A701V
GAMMA	L18F T20N D138Y R190S T1027I
DELTA	Del156-157 R158G
EPSILON	S13I W152C
ETA	Q52R A67V Q677H F888L
THETA	E1092K H1101Y
IOTA	L5F D253G
KAPPA	E154K Q1071H
LAMBDA	T76I R246N Del247-252 F490S T859N
MU	Ins147N Y147N R346K
20 A.EU1	A222V
A 23.1	R102I F157L V367F
B.1.1.318	T95I
OMICRON BA.1	Del143-144 N211I L212V Ins213-214 V215P G446S G449S T547K N856K L981F
OMICRON BA.2.12.1	S704L
OMICRON BA.4	V3G

## Data Availability

Not applicable.

## Supplementary Materials

The following supporting information can be downloaded at: https://www.mdpi.com/article/10.3390/life12081245/s1, Figure S1: Sequences alignment. SARS-CoV-2 wild type spike protein and its most diffused variants were aligned by using the Clustal Omega program (https://www.ebi.ac.uk/Tools/msa/clustalo/, accessed on 1 October 2019). The parameters used were used by default of the program. In yellow are underlined wild type residues with high rate of mutation. In red are underlined mutations present only in a single variant. In green are underlined mutations present only in all the Omicron variants. In cyan are underlined mutations present in all the Omicron variants excluding Omicron BA.1.
