# Supplementary material for: Structural and Phylogenetic Analysis of SARS-CoV-2 Spike Glycoprotein from the Most Widespread Variants
Source: Life (Basel). 2022 Aug 16;12(8):1245. doi: 10.3390/life12081245 (PMC9410480; doi:10.3390/life12081245)
Supplement: Supplementary file 1 [file life-12-01245-s001.zip › life-1838946-supplementary.pdf]

```
wild type              MFVFLVLLPLVSSQCVNLTTRTQLPPAYTNSFTRGVYYPDKVFRSSVLHSTQDLFLPFFS  60
DELTA                  MFVFLVLLPLVSSQCVNLRTRTQLPPAYTNSFTRGVYYPDKVFRSSVLHSTQDLFLPFFS  60
LAMBDA                 MFVFLVLLPLVSSQCVNLTTRTQLPPAYTNSFTRGVYYPDKVFRSSVLHSTQDLFLPFFS  60
MU                     MFVFLVLLPLVSSQCVNLTTRTQLPPAYTNSFTRGVYYPDKVFRSSVLHSTQDLFLPFFS  60
BETA                   MFVFLVLLPLVSSQCVNLTTRTQLPPAYTNSFTRGVYYPDKVFRSSVLHSTQDLFLPFFS  60
GAMMA                  MFVFLVLLPLVSSQCVNFTNRTQLPSAYTNSFTRGVYYPDKVFRSSVLHSTQDLFLPFFS  60
B.1.1.318              MFVFLVLLPLVSSQCVNLTTRTQLPPAYTNSFTRGVYYPDKVFRSSVLHSTQDLFLPFFS  60
KAPPA                  MFVFLVLLPLVSSQCVNLTTRTQLPPAYTNSFTRGVYYPDKVFRSSVLHSTQDLFLPFFS  60
A 23.1                 MFVFLVLLPLVSSQCVNLTTRTQLPPAYTNSFTRGVYYPDKVFRSSVLHSTQDLFLPFFS  60
IOTA                   MFVFFVLLPLVSSQCVNLTTRTQLPPAYTNSFTRGVYYPDKVFRSSVLHSTQDLFLPFFS  60
THETA                  MFVFLVLLPLVSSQCVNLTTRTQLPPAYTNSFTRGVYYPDKVFRSSVLHSTQDLFLPFFS  60
EPSILON                MFVFLVLLPLVSIQCVNLTTRTQLPPAYTNSFTRGVYYPDKVFRSSVLHSTQDLFLPFFS  60
20 A.EU1               MFVFLVLLPLVSSQCVNLTTRTQLPPAYTNSFTRGVYYPDKVFRSSVLHSTQDLFLPFFS  60
20 A.EU2               MFVFLVLLPLVSSQCVNLTTRTQLPPAYTNSFTRGVYYPDKVFRSSVLHSTQDLFLPFFS  60
ZETA                   MFVFLVLLPLVSSQCVNLTTRTQLPPAYTNSFTRGVYYPDKVFRSSVLHSTQDLFLPFFS  60
ALPHA                  MFVFLVLLPLVSSQCVNLTTRTQLPPAYTNSFTRGVYYPDKVFRSSVLHSTQDLFLPFFS  60
ETA                    MFVFLVLLPLVSSQCVNLTTRTQLPPAYTNSFTRGVYYPDKVFRSSVLHSTRDLFLPFFS  60
Omicron BA.1           MFVFLVLLPLVSSQCVNLTTRTQLPPAYTNSFTRGVYYPDKVFRSSVLHSTQDLFLPFFS  60
Omicron BA.2           MFVFLVLLPLVSSQCVNLITRTQ---SYTNSFTRGVYYPDKVFRSSVLHSTQDLFLPFFS  57
Omicron BA.2.12.1      MFVFLVLLPLVSSQCVNLITRTQ---SYTNSFTRGVYYPDKVFRSSVLHSTQDLFLPFFS  57
Omicron BA.4           MFGFLVLLPLVSSQCVNLITRTQ---SYTNSFTRGVYYPDKVFRSSVLHSTQDLFLPFFS  57
Omicron BA.5           MFVFLVLLPLVSSQCVNLITRTQ---SYTNSFTRGVYYPDKVFRSSVLHSTQDLFLPFFS  57
                       ** *:******* ****: .***   :************************:********

wild type              NVTWFHAIHVSGTNGTKRFDNPVLPFNDGVYFASTEKSNIIRGWIFGTTLDSKTQSLLIV  120
DELTA                  NVTWFHAIHVSGTNGTKRFDNPVLPFNDGVYFASTEKSNIIRGWIFGTTLDSKTQSLLIV  120
LAMBDA                 NVTWFHAIHVSGTNVIKRFDNPVLPFNDGVYFASTEKSNIIRGWIFGTTLDSKTQSLLIV  120
MU                     NVTWFHAIHVSGTNGTKRFDNPVLPFNDGVYFASIEKSNIIRGWIFGTTLDSKTQSLLIV  120
BETA                   NVTWFHAIHVSGTNGTKRFANPVLPFNDGVYFASTEKSNIIRGWIFGTTLDSKTQSLLIV  120
GAMMA                  NVTWFHAIHVSGTNGTKRFDNPVLPFNDGVYFASTEKSNIIRGWIFGTTLDSKTQSLLIV  120
B.1.1.318              NVTWFHAIHVSGTNGTKRFDNPVLPFNDGVYFASIEKSNIIRGWIFGTTLDSKTQSLLIV  120
KAPPA                  NVTWFHAIHVSGTNGTKRFDNPVLPFNDGVYFASTEKSNIIRGWIFGTTLDSKTQSLLIV  120
A 23.1                 NVTWFHAIHVSGTNGTKRFDNPVLPFNDGVYFASTEKSNIIIGWIFGTTLDSKTQSLLIV  120
IOTA                   NVTWFHAIHVSGTNGTKRFDNPVLPFNDGVYFASIEKSNIIRGWIFGTTLDSKTQSLLIV  120
THETA                  NVTWFHAIHVSGTNGTKRFDNPVLPFNDGVYFASTEKSNIIRGWIFGTTLDSKTQSLLIV  120
EPSILON                NVTWFHAIHVSGTNGTKRFDNPVLPFNDGVYFASTEKSNIIRGWIFGTTLDSKTQSLLIV  120
20 A.EU1               NVTWFHAIHVSGTNGTKRFDNPVLPFNDGVYFASTEKSNIIRGWIFGTTLDSKTQSLLIV  120
20 A.EU2               NVTWFHAIHVSGTNGTKRFDNPVLPFNDGVYFASTEKSNIIRGWIFGTTLDSKTQSLLIV  120
ZETA                   NVTWFHAIHVSGTNGTKRFDNPVLPFNDGVYFASTEKSNIIRGWIFGTTLDSKTQSLLIV  120
ALPHA                  NVTWFHAI--SGTNGTKRFDNPVLPFNDGVYFASTEKSNIIRGWIFGTTLDSKTQSLLIV  118
ETA                    NVTWFHVI--SGTNGTKRFDNPVLPFNDGVYFASTEKSNIIRGWIFGTTLDSKTQSLLIV  118
Omicron BA.1           NVTWFHVI--SGTNGTKRFDNPVLPFNDGVYFASIEKSNIIRGWIFGTTLDSKTQSLLIV  118
Omicron BA.2           NVTWFHAIHVSGTNGTKRFDNPVLPFNDGVYFASTEKSNIIRGWIFGTTLDSKTQSLLIV  117
Omicron BA.2.12.1      NVTWFHAIHVSGTNGTKRFDNPVLPFNDGVYFASTEKSNIIRGWIFGTTLDSKTQSLLIV  117
Omicron BA.4           NVTWFHAI--SGTNGTKRFDNPVLPFNDGVYFASTEKSNIIRGWIFGTTLDSKTQSLLIV  115
Omicron BA.5           NVTWFHAI--SGTNGIKRFDNPVLPFNDGVYFASTEKSNIIRGWIFGTTLDSKTQSLLIV  115
                       ******.*  ****  *** ************** ****** ******************

wild type              NNATNVVIKVCEFQFCNDPFLGVY-YHKNNKSWMESEFRVYSSANNCTFEYVSQPFLMDL  179
DELTA                  NNATNVVIKVCEFQFCNDPFLDVY-YHKNNKSWMES--GVYSSANNCTFEYVSQPFLMDL  177
LAMBDA                 NNATNVVIKVCEFQFCNDPFLGVY-YHKNNKSWMESEFRVYSSANNCTFEYVSQPFLMDL  179
MU                     NNATNVVIKVCEFQFCNDPFLGVSNNHKNNKSWMESEFRVYSSANNCTFEYVSQPFLMDL  180
BETA                   NNATNVVIKVCEFQFCNDPFLGVY-YHKNNKSWMESEFRVYSSANNCTFEYVSQPFLMDL  179
GAMMA                  NNATNVVIKVCEFQFCNYPFLGVY-YHKNNKSWMESEFRVYSSANNCTFEYVSQPFLMDL  179
B.1.1.318              NNATNVVIKVCEFQFCNDPFLGVY--HKNNKSWMESEFRVYSSANNCTFEYVSQPFLMDL  178
KAPPA                  NNATNVVIKVCEFQFCNDPFLGVY-YHKNNKSWMKSEFRVYSSANNCTFEYVSQPFLMDL  179
A 23.1                 NNATNVVIKVCEFQFCNDPFLGVY-YHKNNKSWMESELRVYSSANNCTFEYVSQPFLMDL  179
IOTA                   NNATNVVIKVCEFQFCNDPFLGVY-YHKNNKSWMESEFRVYSSANNCTFEYVSQPFLMDL  179
THETA                  NNATNVVIKVCEFQFCNDPFLGVY-YHKNNKSWMESEFRVYSSANNCTFEYVSQPFLMDL  179
EPSILON                NNATNVVIKVCEFQFCNDPFLGVY-YHKNNKSCMESEFRVYSSANNCTFEYVSQPFLMDL  179
20 A.EU1               NNATNVVIKVCEFQFCNDPFLGVY-YHKNNKSWMESEFRVYSSANNCTFEYVSQPFLMDL  179
20 A.EU2               NNATNVVIKVCEFQFCNDPFLGVY-YHKNNKSWMESEFRVYSSANNCTFEYVSQPFLMDL  179
ZETA                   NNATNVVIKVCEFQFCNDPFLGVY-YHKNNKSWMESEFRVYSSANNCTFEYVSQPFLMDL  179
ALPHA                  NNATNVVIKVCEFQFCNDPFLGV--YHKNNKSWMESEFRVYSSANNCTFEYVSQPFLMDL  176
ETA                    NNATNVVIKVCEFQFCNDPFLGV--YHKNNKSWMESEFRVYSSANNCTFEYVSQPFLMDL  176
Omicron BA.1           NNATNVVIKVCEFQFCNDPFLD----HKNNKSWMESEFRVYSSANNCTFEYVSQPFLMDL  174
Omicron BA.2           NNATNVVIKVCEFQFCNDPFLDVY-YHKNNKSWMESEFRVYSSANNCTFEYVSQPFLMDL  176
Omicron BA.2.12.1      NNATNVVIKVCEFQFCNDPFLDVY-YHKNNKSWMESEFRVYSSANNCTFEYVSQPFLMDL  176
Omicron BA.4           NNATNVVIKVCEFQFCNDPFLDVY-YHKNNKSWMESEFRVYSSANNCTFEYVSQPFLMDL  174
Omicron BA.5           NNATNVVIKVCEFQFCNDPFLDVY-YHKNNKSWMESEFRVYSSANNCTFEYVSQPFLMDL  174
                       ***************** ***.    ****** *:*   **********************
```

```
wild type              EGKQGNFKNLREFVFKNIDGYFKIYSKHTPINL--VRDLPQGFSALEPLVDLPIGINITR  237
DELTA                  EGKQGNFKNLREFVFKNIDGYFKIYSKHTPINL--VRDLPQGFSALEPLVDLPIGINITR  235
LAMBDA                 EGKQGNFKNLREFVFKNIDGYFKIYSKHTPINL--VRDLPQGFSALEPLVDLPIGINITR  237
MU                     EGKQGNFKNLREFVFKNIDGYFKIYSKHTPINL--VRDLPQGFSALEPLVDLPIGINITR  238
BETA                   EGKQGNFKNLREFVFKNIDGYFKIYSKHTPINL--VRGLPQGFSALEPLVDLPIGINITR  237
GAMMA                  EGKQGNFKNLSEFVFKNIDGYFKIYSKHTPINL--VRDLPQGFSALEPLVDLPIGINITR  237
B.1.1.318              EGKQGNFKNLREFVFKNIDGYFKIYSKHTPINL--VRDLPQGFSALEPLVDLPIGINITR  236
KAPPA                  EGKQGNFKNLREFVFKNIDGYFKIYSKHTPINL--VRDLPQGFSALEPLVDLPIGINITR  237
A 23.1                 EGKQGNFKNLREFVFKNIDGYFKIYSKHTPINL--VRDLPQGFSALEPLVDLPIGINITR  237
IOTA                   EGKQGNFKNLREFVFKNIDGYFKIYSKHTPINL--VRDLPQGFSALEPLVDLPIGINITR  237
THETA                  EGKQGNFKNLREFVFKNIDGYFKIYSKHTPINL--VRDLPQGFSALEPLVDLPIGINITR  237
EPSILON                EGKQGNFKNLREFVFKNIDGYFKIYSKHTPINL--VRDLPQGFSALEPLVDLPIGINITR  237
20 A.EU1               EGKQGNFKNLREFVFKNIDGYFKIYSKHTPINL--VRDLPQGFSVLEPLVDLPIGINITR  237
20 A.EU2               EGKQGNFKNLREFVFKNIDGYFKIYSKHTPINL--VRDLPQGFSALEPLVDLPIGINITR  237
ZETA                   EGKQGNFKNLREFVFKNIDGYFKIYSKHTPINL--VRDLPQGFSALEPLVDLPIGINITR  237
ALPHA                  EGKQGNFKNLREFVFKNIDGYFKIYSKHTPINL--VRDLPQGFSALEPLVDLPIGINITR  234
ETA_                   EGKQGNFKNLREFVFKNIDGYFKIYSKHTPINL--VRDLPQGFSALEPLVDLPIGINITR  234
Omicron BA.1           EGKQGNFKNLREFVFKNIDGYFKIYSKHTPIIVREPEDLPQGFSALEPLVDLPIGINITR  234
Omicron BA.2           EGKQGNFKNLREFVFKNIDGYFKIYSKHTPINL--GRDLPQGFSALEPLVDLPIGINITR  234
Omicron BA.2.12.1      EGKQGNFKNLREFVFKNIDGYFKIYSKHTPINL--GRDLPQGFSALEPLVDLPIGINITR  234
Omicron BA.4           EGKQGNFKNLREFVFKNIDGYFKIYSKHTPINL--GRDLPQGFSALEPLVDLPIGINITR  232
Omicron BA.5           EGKQGNFKNLREFVFKNIDGYFKIYSKHTPINL--GRDLPQGFSALEPLVDLPIGINITR  232
                       ********** ******************** :   ..******.***************
```

```
wild type              FQTLLALHRSYLTPGDSSSGWTAGAAAYYVGYLQPRTFLLKYNENGTITDAVDCALDPLS  297
DELTA                  FQTLLALHRSYLTPGDSSSGWTAGAAAYYVGYLQPRTFLLKYNENGTITDAVDCALDPLS  295
LAMBDA                 FQTLLALHN-------SSSGWTAGAAAYYVGYLQPRTFLLKYNENGTITDAVDCALDPLS  290
MU                     FQTLLALHRSYLTPGDSSSGWTAGAAAYYVGYLQPRTFLLKYNENGTITDAVDCALDPLS  298
BETA                   FQT---LHRSYLTPGDSSSGWTAGAAAYYVGYLQPRTFLLKYNENGTITDAVDCALDPLS  294
GAMMA                  FQTLLALHRSYLTPGDSSSGWTAGAAAYYVGYLQPRTFLLKYNENGTITDAVDCALDPLS  297
B.1.1.318              FQTLLALHRSYLTPGDSSSGWTAGAAAYYVGYLQPRTFLLKYNENGTITDAVDCALDPLS  296
KAPPA                  FQTLLALHRSYLTPGDSSSGWTAGAAAYYVGYLQPRTFLLKYNENGTITDAVDCALDPLS  297
A 23.1                 FQTLLALHRSYLTPGDSSSGWTAGAAAYYVGYLQPRTFLLKYNENGTITDAVDCALDPLS  297
IOTA                   FQTLLALHRSYLTPGGSSSGWTAGAAAYYVGYLQPRTFLLKYNENGTITDAVDCALDPLS  297
THETA                  FQTLLALHRSYLTPGDSSSGWTAGAAAYYVGYLQPRTFLLKYNENGTITDAVDCALDPLS  297
EPSILON                FQTLLALHRSYLTPGDSSSGWTAGAAAYYVGYLQPRTFLLKYNENGTITDAVDCALDPLS  297
20 A.EU1               FQTLLALHRSYLTPGDSSSGWTAGAAAYYVGYLQPRTFLLKYNENGTITDAVDCALDPLS  297
20 A.EU2               FQTLLALHRSYLTPGDSSSGWTAGAAAYYVGYLQPRTFLLKYNENGTITDAVDCALDPLS  297
ZETA                   FQTLLALHRSYLTPGDSSSGWTAGAAAYYVGYLQPRTFLLKYNENGTITDAVDCALDPLS  297
ALPHA                  FQTLLALHRSYLTPGDSSSGWTAGAAAYYVGYLQPRTFLLKYNENGTITDAVDCALDPLS  294
ETA                    FQTLLALHRSYLTPGDSSSGWTAGAAAYYVGYLQPRTFLLKYNENGTITDAVDCALDPLS  294
Omicron BA.1           FQTLLALHRSYLTPGDSSSGWTAGAAAYYVGYLQPRTFLLKYNENGTITDAVDCALDPLS  294
Omicron BA.2           FQTLLALHRSYLTPGDSSSGWTAGAAAYYVGYLQPRTFLLKYNENGTITDAVDCALDPLS  294
Omicron BA.2.12.1      FQTLLALHRSYLTPGDSSSGWTAGAAAYYVGYLQPRTFLLKYNENGTITDAVDCALDPLS  294
Omicron BA.4           FQTLLALHRSYLTPGDSSSGWTAGAAAYYVGYLQPRTFLLKYNENGTITDAVDCALDPLS  292
Omicron BA.5           FQTLLALHRSYLTPGDSSSGWTAGAAAYYVGYLQPRTFLLKYNENGTITDAVDCALDPLS  292
                       ***   **.       ********************************************
```

```
wild type              ETKCTLKSFTVEKGIYQTSNFRVQPTESIVRFPNITNLCPFGEVFNATRFASVYAWNRKR  357
DELTA                  ETKCTLKSFTVEKGIYQTSNFRVQPTESIVRFPNITNLCPFGEVFNATRFASVYAWNRKR  355
LAMBDA                 ETKCTLKSFTVEKGIYQTSNFRVQPTESIVRFPNITNLCPFGEVFNATRFASVYAWNRKR  350
MU                     ETKCTLKSFTVEKGIYQTSNFRVQPTESIVRFPNITNLCPFGEVFNATKFASVYAWNRKR  358
BETA                   ETKCTLKSFTVEKGIYQTSNFRVQPTESIVRFPNITNLCPFGEVFNATRFASVYAWNRKR  354
GAMMA                  ETKCTLKSFTVEKGIYQTSNFRVQPTESIVRFPNITNLCPFGEVFNATRFASVYAWNRKR  357
B.1.1.318              ETKCTLKSFTVEKGIYQTSNFRVQPTESIVRFPNITNLCPFGEVFNATRFASVYAWNRKR  356
KAPPA                  ETKCTLKSFTVEKGIYQTSNFRVQPTESIVRFPNITNLCPFGEVFNATRFASVYAWNRKR  357
A 23.1                 ETKCTLKSFTVEKGIYQTSNFRVQPTESIVRFPNITNLCPFGEVFNATRFASVYAWNRKR  357
IOTA                   ETKCTLKSFTVEKGIYQTSNFRVQPTESIVRFPNITNLCPFGEVFNATRFASVYAWNRKR  357
THETA                  ETKCTLKSFTVEKGIYQTSNFRVQPTESIVRFPNITNLCPFGEVFNATRFASVYAWNRKR  357
EPSILON                ETKCTLKSFTVEKGIYQTSNFRVQPTESIVRFPNITNLCPFGEVFNATRFASVYAWNRKR  357
20 A.EU1               ETKCTLKSFTVEKGIYQTSNFRVQPTESIVRFPNITNLCPFGEVFNATRFASVYAWNRKR  357
20 A.EU2               ETKCTLKSFTVEKGIYQTSNFRVQPTESIVRFPNITNLCPFGEVFNATRFASVYAWNRKR  357
ZETA                   ETKCTLKSFTVEKGIYQTSNFRVQPTESIVRFPNITNLCPFGEVFNATRFASVYAWNRKR  357
ALPHA                  ETKCTLKSFTVEKGIYQTSNFRVQPTESIVRFPNITNLCPFGEVFNATRFASVYAWNRKR  354
ETA                    ETKCTLKSFTVEKGIYQTSNFRVQPTESIVRFPNITNLCPFGEVFNATRFASVYAWNRKR  354
Omicron BA.1           ETKCTLKSFTVEKGIYQTSNFRVQPTESIVRFPNITNLCPFDEVFNATRFASVYAWNRKR  354
Omicron BA.2           ETKCTLKSFTVEKGIYQTSNFRVQPTESIVRFPNITNLCPFDEVFNATRFASVYAWNRKR  354
Omicron BA.2.12.1      ETKCTLKSFTVEKGIYQTSNFRVQPTESIVRFPNITNLCPFDEVFNATRFASVYAWNRKR  354
Omicron BA.4           ETKCTLKSFTVEKGIYQTSNFRVQPTESIVRFPNITNLCPFDEVFNATRFASVYAWNRKR  352
Omicron BA.5           ETKCTLKSFTVEKGIYQTSNFRVQPTESIVRFPNITNLCPFDEVFNATRFASVYAWNRKR  352
                       *****************************************.******:***********
```

```
wild type          ISNCVADYSVLYNSASFSTFKCYGVSPTKLNDLCFTNVYADSFVIRGDEVRQIAPGQTGK  417
DELTA              ISNCVADYSVLYNSASFSTFKCYGVSPTKLNDLCFTNVYADSFVIRGDEVRQIAPGQTGK  415
LAMBDA             ISNCVADYSVLYNSASFSTFKCYGVSPTKLNDLCFTNVYADSFVIRGDEVRQIAPGQTGK  410
MU                 ISNCVADYSVLYNSASFSTFKCYGVSPTKLNDLCFTNVYADSFVIRGDEVRQIAPGQTGK  418
BETA               ISNCVADYSVLYNSASFSTFKCYGVSPTKLNDLCFTNVYADSFVIRGDEVRQIAPGQTGN  414
GAMMA              ISNCVADYSVLYNSASFSTFKCYGVSPTKLNDLCFTNVYADSFVIRGDEVRQIAPGQTGT  417
B.1.1.318          ISNCVADYSVLYNSASFSTFKCYGVSPTKLNDLCFTNVYADSFVIRGDEVRQIAPGQTGK  416
KAPPA              ISNCVADYSVLYNSASFSTFKCYGVSPTKLNDLCFTNVYADSFVIRGDEVRQIAPGQTGK  417
A 23.1             ISNCVADYSFLYNSASFSTFKCYGVSPTKLNDLCFTNVYADSFVIRGDEVRQIAPGQTGK  417
IOTA               ISNCVADYSVLYNSASFSTFKCYGVSPTKLNDLCFTNVYADSFVIRGDEVRQIAPGQTGK  417
THETA              ISNCVADYSVLYNSASFSTFKCYGVSPTKLNDLCFTNVYADSFVIRGDEVRQIAPGQTGK  417
EPSILON            ISNCVADYSVLYNSASFSTFKCYGVSPTKLNDLCFTNVYADSFVIRGDEVRQIAPGQTGK  417
20 A.EU1           ISNCVADYSVLYNSASFSTFKCYGVSPTKLNDLCFTNVYADSFVIRGDEVRQIAPGQTGK  417
20 A.EU2           ISNCVADYSVLYNSASFSTFKCYGVSPTKLNDLCFTNVYADSFVIRGDEVRQIAPGQTGK  417
ZETA               ISNCVADYSVLYNSASFSTFKCYGVSPTKLNDLCFTNVYADSFVIRGDEVRQIAPGQTGK  417
ALPHA              ISNCVADYSVLYNSASFSTFKCYGVSPTKLNDLCFTNVYADSFVIRGDEVRQIAPGQTGK  414
ETA                ISNCVADYSVLYNSASFSTFKCYGVSPTKLNDLCFTNVYADSFVIRGDEVRQIAPGQTGK  414
Omicron BA.1       ISNCVADYSVLYNLAPFFTFKCYGVSPTKLNDLCFTNVYADSFVIRGDEVRQIAPGQTGN  414
Omicron BA.2       ISNCVADYSVLYNFAPFFAFKCYGVSPTKLNDLCFTNVYADSFVIRGNEVSQIAPGQTGN  414
Omicron BA.2.12.1  ISNCVADYSVLYNFAPFFAFKCYGVSPTKLNDLCFTNVYADSFVIRGNEVSQIAPGQTGN  414
Omicron BA.4       ISNCVADYSVLYNFAPFFAFKCYGVSPTKLNDLCFTNVYADSFVIRGNEVSQIAPGQTGN  412
Omicron BA.5       ISNCVADYSVLYNFAPFFAFKCYGVSPTKLNDLCFTNVYADSFVIRGNEVSQIAPGQTGN  412
                   *********.*** * * :****************************:** ********.

wild type          IADYNYKLPDDFTGCVIAWNSNNLDSKVGGNYNYLYRLFRKSNLKPFERDISTEIYQAGS  477
DELTA              IADYNYKLPDDFTGCVIAWNSNNLDSKVGGNYNYRYRLFRKSNLKPFERDISTEIYQAGS  475
LAMBDA             IADYNYKLPDDFTGCVIAWNSNNLDSKVGGNYNYQYRLFRKSNLKPFERDISTEIYQAGS  470
MU                 IADYNYKLPDDFTGCVIAWNSNNLDSKVGGNYNYLYRLFRKSNLKPFERDISTEIYQAGS  478
BETA               IADYNYKLPDDFTGCVIAWNSNNLDSKVGGNYNYLYRLFRKSNLKPFERDISTEIYQAGS  474
GAMMA              IADYNYKLPDDFTGCVIAWNSNNLDSKVGGNYNYLYRLFRKSNLKPFERDISTEIYQAGS  477
B.1.1.318          IADYNYKLPDDFTGCVIAWNSNNLDSKVGGNYNYLYRLFRKSNLKPFERDISTEIYQAGS  476
KAPPA              IADYNYKLPDDFTGCVIAWNSNNLDSKVGGNYNYRYRLFRKSNLKPFERDISTEIYQAGS  477
A 23.1             IADYNYKLPDDFTGCVIAWNSNNLDSKVGGNYNYLYRLFRKSNLKPFERDISTEIYQAGS  477
IOTA               IADYNYKLPDDFTGCVIAWNSNNLDSKVGGNYNYLYRLFRKSNLKPFERDISTEIYQAGS  477
THETA              IADYNYKLPDDFTGCVIAWNSNNLDSKVGGNYNYLYRLFRKSNLKPFERDISTEIYQAGS  477
EPSILON            IADYNYKLPDDFTGCVIAWNSNNLDSKVGGNYNYRYRLFRKSNLKPFERDISTEIYQAGS  477
20 A.EU1           IADYNYKLPDDFTGCVIAWNSNNLDSKVGGNYNYLYRLFRKSNLKPFERDISTEIYQAGS  477
20 A.EU2           IADYNYKLPDDFTGCVIAWNSNNLDSKVGGNYNYLYRLFRKSNLKPFERDISTEIYQAGN  477
ZETA               IADYNYKLPDDFTGCVIAWNSNNLDSKVGGNYNYLYRLFRKSNLKPFERDISTEIYQAGS  477
ALPHA              IADYNYKLPDDFTGCVIAWNSNNLDSKVGGNYNYLYRLFRKSNLKPFERDISTEIYQAGS  474
ETA                IADYNYKLPDDFTGCVIAWNSNNLDSKVGGNYNYLYRLFRKSNLKPFERDISTEIYQAGS  474
Omicron BA.1       IADYNYKLPDDFTGCVIAWNSNKLDSKVSGNYNYLYRLFRKSNLKPFERDISTEIYQAGN  474
Omicron BA.2       IADYNYKLPDDFTGCVIAWNSNKLDSKVGGNYNYLYRLFRKSNLKPFERDISTEIYQAGN  474
Omicron BA.2.12.1  IADYNYKLPDDFTGCVIAWNSNKLDSKVGGNYNYQYRLFRKSNLKPFERDISTEIYQAGN  474
Omicron BA.4       IADYNYKLPDDFTGCVIAWNSNKLDSKVGGNYNYRYRLFRKSNLKPFERDISTEIYQAGN  472
Omicron BA.5       IADYNYKLPDDFTGCVIAWNSNKLDSKVGGNYNYRYRLFRKSNLKPFERDISTEIYQAGN  472
                   **********************:*****.***** ************************.

wild type          TPCNGVEGFNCYFPLQSYGFQPTNGVGYQPYRVVVLSFELLHAPATVCGPKKSTNLVKNK  537
DELTA              KPCNGVEGFNCYFPLQSYGFQPTNGVGYQPYRVVVLSFELLHAPATVCGPKKSTNLVKNK  535
LAMBDA             TPCNGVEGFNCYSPLQSYGFQPTNGVGYQPYRVVVLSFELLHAPATVCGPKKSTNLVKNK  530
MU                 TPCNGVKGFNCYFPLQSYGFQPTYGVGYQPYRVVVLSFELLHAPATVCGPKKSTNLVKNK  538
BETA               TPCNGVKGFNCYFPLQSYGFQPTYGVGYQPYRVVVLSFELLHAPATVCGPKKSTNLVKNK  534
GAMMA              TPCNGVKGFNCYFPLQSYGFQPTYGVGYQPYRVVVLSFELLHAPATVCGPKKSTNLVKNK  537
B.1.1.318          TPCNGVEGFNCYFPLQSYGFQPTNGVGYQPYRVVVLSFELLHAPATVCGPKKSTNLVKNK  536
KAPPA              TPCNGVQGFNCYFPLQSYGFQPTNGVGYQPYRVVVLSFELLHAPATVCGPKKSTNLVKNK  537
A 23.1             TPCNGVEGFNCYFPLQSYGFQPTNGVGYQPYRVVVLSFELLHAPATVCGPKKSTNLVKNK  537
IOTA               TPCNGVKGFNCYFPLQSYGFQPTNGVGYQPYRVVVLSFELLHAPATVCGPKKSTNLVKNK  537
THETA              TPCNGVKGFNCYFPLQSYGFQPTYGVGYQPYRVVVLSFELLHAPATVCGPKKSTNLVKNK  537
EPSILON            TPCNGVEGFNCYFPLQSYGFQPTNGVGYQPYRVVVLSFELLHAPATVCGPKKSTNLVKNK  537
20 A.EU1           TPCNGVEGFNCYFPLQSYGFQPTNGVGYQPYRVVVLSFELLHAPATVCGPKKSTNLVKNK  537
20 A.EU2           TPCNGVEGFNCYFPLQSYGFQPTNGVGYQPYRVVVLSFELLHAPATVCGPKKSTNLVKNK  537
ZETA               TPCNGVKGFNCYFPLQSYGFQPTNGVGYQPYRVVVLSFELLHAPATVCGPKKSTNLVKNK  537
ALPHA              TPCNGVEGFNCYFPLQSYGFQPTYGVGYQPYRVVVLSFELLHAPATVCGPKKSTNLVKNK  534
ETA                TPCNGVKGFNCYFPLQSYGFQPTNGVGYQPYRVVVLSFELLHAPATVCGPKKSTNLVKNK  534
Omicron BA.1       KPCNGVAGFNCYFPLRSYSFRPTYGVGHQPYRVVVLSFELLHAPATVCGPKKSTNLVKNK  534
Omicron BA.2       KPCNGVAGFNCYFPLRSYGFRPTYGVGHQPYRVVVLSFELLHAPATVCGPKKSTNLVKNK  534
Omicron BA.2.12.1  KPCNGVAGFNCYFPLRSYGFRPTYGVGHQPYRVVVLSFELLHAPATVCGPKKSTNLVKNK  534
Omicron BA.4       KPCNGVAGVNCYFPLQSYGFRPTYGVGHQPYRVVVLSFELLHAPATVCGPKKSTNLVKNK  532
Omicron BA.5       KPCNGVAGVNCYFPLQSYGFRPTYGVGHQPYRVVVLSFELLHAPATVCGPKKSTNLVKNK  532
                   .***** *.*** **:**.*:** ***:********************************
```

```
wild type           CVNFNFNGLTGTGVLTESNKKFLPFQQFGRDIADTTDAVRDPQTLEILDITPCSFGGVSV  597
DELTA               CVNFNFNGLTGTGVLTESNKKFLPFQQFGRDIADTTDAVRDPQTLEILDITPCSFGGVSV  595
LAMBDA              CVNFNFNGLTGTGVLTESNKKFLPFQQFGRDIADTTDAVRDPQTLEILDITPCSFGGVSV  590
MU                  CVNFNFNGLTGTGVLTESNKKFLPFQQFGRDIADTTDAVRDPQTLEILDITPCSFGGVSV  598
BETA                CVNFNFNGLTGTGVLTESNKKFLPFQQFGRDIADTTDAVRDPQTLEILDITPCSFGGVSV  594
GAMMA               CVNFNFNGLTGTGVLTESNKKFLPFQQFGRDIADTTDAVRDPQTLEILDITPCSFGGVSV  597
B.1.1.318           CVNFNFNGLTGTGVLTESNKKFLPFQQFGRDIADTTDAVRDPQTLEILDITPCSFGGVSV  596
KAPPA               CVNFNFNGLTGTGVLTESNKKFLPFQQFGRDIADTTDAVRDPQTLEILDITPCSFGGVSV  597
A 23.1              CVNFNFNGLTGTGVLTESNKKFLPFQQFGRDIADTTDAVRDPQTLEILDITPCSFGGVSV  597
IOTA                CVNFNFNGLTGTGVLTESNKKFLPFQQFGRDIADTTDAVRDPQTLEILDITPCSFGGVSV  597
THETA               CVNFNFNGLTGTGVLTESNKKFLPFQQFGRDIADTTDAVRDPQTLEILDITPCSFGGVSV  597
EPSILON             CVNFNFNGLTGTGVLTESNKKFLPFQQFGRDIADTTDAVRDPQTLEILDITPCSFGGVSV  597
20 A.EU1            CVNFNFNGLTGTGVLTESNKKFLPFQQFGRDIADTTDAVRDPQTLEILDITPCSFGGVSV  597
20 A.EU2            CVNFNFNGLTGTGVLTESNKKFLPFQQFGRDIADTTDAVRDPQTLEILDITPCSFGGVSV  597
ZETA                CVNFNFNGLTGTGVLTESNKKFLPFQQFGRDIADTTDAVRDPQTLEILDITPCSFGGVSV  597
ALPHA               CVNFNFNGLTGTGVLTESNKKFLPFQQFGRDIDDTTDAVRDPQTLEILDITPCSFGGVSV  594
ETA                 CVNFNFNGLTGTGVLTESNKKFLPFQQFGRDIADTTDAVRDPQTLEILDITPCSFGGVSV  594
Omicron BA.1        CVNFNFNGLKGTGVLTESNKKFLPFQQFGRDIADTTDAVRDPQTLEILDITPCSFGGVSV  594
Omicron BA.2        CVNFNFNGLTGTGVLTESNKKFLPFQQFGRDIADTTDAVRDPQTLEILDITPCSFGGVSV  594
Omicron BA.2.12.1   CVNFNFNGLTGTGVLTESNKKFLPFQQFGRDIADTTDAVRDPQTLEILDITPCSFGGVSV  594
Omicron BA.4        CVNFNFNGLTGTGVLTESNKKFLPFQQFGRDIADTTDAVRDPQTLEILDITPCSFGGVSV  592
Omicron BA.5        CVNFNFNGLTGTGVLTESNKKFLPFQQFGRDIADTTDAVRDPQTLEILDITPCSFGGVSV  592
                    *********.********************** ***************************


wild type           ITPGTNTSNQVAVLYQDVNCTEVPVAIHADQLTPTWRVYSTGSNVFQTRAGCLIGAEHVN  657
DELTA               ITPGTNTSNQVAVLYQGVNCTEVPVAIHADQLTPTWRVYSTGSNVFQTRAGCLIGAEHVN  655
LAMBDA              ITPGTNTSNQVAVLYQGVNCTEVPVAIHADQLTPTWRVYSTGSNVFQTRAGCLIGAEHVN  650
MU                  ITPGTNTSNQVAVLYQGVNCTEVPVAIHADQLTPTWRVYSTGSNVFQTRAGCLIGAEHVN  658
BETA                ITPGTNTSNQVAVLYQGVNCTEVPVAIHADQLTPTWRVYSTGSNVFQTRAGCLIGAEHVN  654
GAMMA               ITPGTNTSNQVAVLYQGVNCTEVPVAIHADQLTPTWRVYSTGSNVFQTRAGCLIGAEYVN  657
B.1.1.318           ITPGTNTSNQVAVLYQHVNCTEVPVAIHADQLTPTWRVYSTGSNVFQTRAGCLIGAEHVN  656
KAPPA               ITPGTNTSNQVAVLYQGVNCTEVPVAIHADQLTPTWRVYSTGSNVFQTRAGCLIGAEHVN  657
A 23.1              ITPGTNTSNQVAVLYHDVNCTEVPVAIHADQLTPTWRVYSTGSNVFQTRAGCLIGAEHVN  657
IOTA                ITPGTNTSNQVAVLYQGVNCTEVPVAIHADQLTPTWRVYSTGSNVFQTRAGCLIGAEHVN  657
THETA               ITPGTNTSNQVAVLYQGVNCTEVPVAIHADQLTPTWRVYSTGSNVFQTRAGCLIGAEHVN  657
EPSILON             ITPGTNTSNQVAVLYQGVNCTEVPVAIHADQLTPTWRVYSTGSNVFQTRAGCLIGAEHVN  657
20 A.EU1            ITPGTNTSNQVAVLYQGVNCTEVPVAIHADQLTPTWRVYSTGSNVFQTRAGCLIGAEHVN  657
20 A.EU2            ITPGTNTSNQVAVLYQGVNCTEVPVAIHADQLTPTWRVYSTGSNVFQTRAGCLIGAEHVN  657
ZETA                ITPGTNTSNQVAVLYQGVNCTEVPVAIHADQLTPTWRVYSTGSNVFQTRAGCLIGAEHVN  657
ALPHA               ITPGTNTSNQVAVLYQGVNCTEVPVAIHADQLTPTWRVYSTGSNVFQTRAGCLIGAEHVN  654
ETA                 ITPGTNTSNQVAVLYQGVNCTEVPVAIHADQLTPTWRVYSTGSNVFQTRAGCLIGAEHVN  654
Omicron BA.1        ITPGTNTSNQVAVLYQGVNCTEVPVAIHADQLTPTWRVYSTGSNVFQTRAGCLIGAEYVN  654
Omicron BA.2        ITPGTNTSNQVAVLYQGVNCTEVPVAIHADQLTPTWRVYSTGSNVFQTRAGCLIGAEYVN  654
Omicron BA.2.12.1   ITPGTNTSNQVAVLYQGVNCTEVPVAIHADQLTPTWRVYSTGSNVFQTRAGCLIGAEYVN  654
Omicron BA.4        ITPGTNTSNQVAVLYQGVNCTEVPVAIHADQLTPTWRVYSTGSNVFQTRAGCLIGAEYVN  652
Omicron BA.5        ITPGTNTSNQVAVLYQGVNCTEVPVAIHADQLTPTWRVYSTGSNVFQTRAGCLIGAEYVN  652
                    ***************: ****************************************:**


wild type           NSYECDIPIGAGICASYQTQTNSPRRARSVASQSIIAYTMSLGAENSVAYSNNSIAIPTN  717
DELTA               NSYECDIPIGAGICASYQTQTNSRRRARSVASQSIIAYTMSLGAENSVAYSNNSIAIPTN  715
LAMBDA              NSYECDIPIGAGICASYQTQTNSPRRARSVASQSIIAYTMSLGAENSVAYSNNSIAIPTN  710
MU                  NSYECDIPIGAGICASYQTQTNSHRRARSVASQSIIAYTMSLGAENSVAYSNNSIAIPTN  718
BETA                NSYECDIPIGAGICASYQTQTNSPRRARSVASQSIIAYTMSLGVENSVAYSNNSIAIPTN  714
GAMMA               NSYECDIPIGAGICASYQTQTNSPRRARSVASQSIIAYTMSLGAENSVAYSNNSIAIPTN  717
B.1.1.318           NSYECDIPIGAGICASYQTQTNSHRRARSVASQSIIAYTMSLGAENSVAYSNNSIAIPTN  716
KAPPA               NSYECDIPIGAGICASYQTQTNSRRRARSVASQSIIAYTMSLGAENSVAYSNNSIAIPTN  717
A 23.1              NSYECDIPIGAGICASYQTQTNSRRRARSVASQSIIAYTMSLGAENSVAYSNNSIAIPTN  717
IOTA                NSYECDIPIGAGICASYQTQTNSPRRARSVASQSIIAYTMSLGVENSVAYSNNSIAIPTN  717
THETA               NSYECDIPIGAGICASYQTQTNSHRRARSVASQSIIAYTMSLGAENSVAYSNNSIAIPTN  717
EPSILON             NSYECDIPIGAGICASYQTQTNSPRRARSVASQSIIAYTMSLGAENSVAYSNNSIAIPTN  717
20 A.EU1            NSYECDIPIGAGICASYQTQTNSPRRARSVASQSIIAYTMSLGAENSVAYSNNSIAIPTN  717
20 A.EU2            NSYECDIPIGAGICASYQTQTNSPRRARSVASQSIIAYTMSLGAENSVAYSNNSIAIPTN  717
ZETA                NSYECDIPIGAGICASYQTQTNSPRRARSVASQSIIAYTMSLGAENSVAYSNNSIAIPTN  717
ALPHA               NSYECDIPIGAGICASYQTQTNSHRRARSVASQSIIAYTMSLGAENSVAYSNNSIAIPIN  714
ETA                 NSYECDIPIGAGICASYQTHTNSPRRARSVASQSIIAYTMSLGAENSVAYSNNSIAIPTN  714
Omicron BA.1        NSYECDIPIGAGICASYQTQTKSHRRARSVASQSIIAYTMSLGAENSVAYSNNSIAIPTN  714
Omicron BA.2        NSYECDIPIGAGICASYQTQTKSHRRARSVASQSIIAYTMSLGAENSVAYSNNSIAIPTN  714
Omicron BA.2.12.1   NSYECDIPIGAGICASYQTQTKSHRRARSVASQSIIAYTMSLGAENLVAYSNNSIAIPTN  714
Omicron BA.4        NSYECDIPIGAGICASYQTQTKSHRRARSVASQSIIAYTMSLGAENSVAYSNNSIAIPTN  712
Omicron BA.5        NSYECDIPIGAGICASYQTQTKSHRRARSVASQSIIAYTMSLGAENSVAYSNNSIAIPTN  712
                    *******************:*:* *******************.** *********** *
```

```
wild type              FTISVTTEILPVSMTKTSVDCTMYICGDSTECSNLLLQYGSFCTQLNRALTGIAVEQDKN  777
DELTA                  FTISVTTEILPVSMTKTSVDCTMYICGDSTECSNLLLQYGSFCTQLNRALTGIAVEQDKN  775
LAMBDA                 FTISVTTEILPVSMTKTSVDCTMYICGDSTECSNLLLQYGSFCTQLNRALTGIAVEQDKN  770
MU                     FTISVTTEILPVSMTKTSVDCTMYICGDSTECSNLLLQYGSFCTQLNRALTGIAVEQDKN  778
BETA                   FTISVTTEILPVSMTKTSVDCTMYICGDSTECSNLLLQYGSFCTQLNRALTGIAVEQDKN  774
GAMMA                  FTISVTTEILPVSMTKTSVDCTMYICGDSTECSNLLLQYGSFCTQLNRALTGIAVEQDKN  777
B.1.1.318              FTISVTTEILPVSMTKTSVDCTMYICGDSTECSNLLLQYGSFCTQLNRALTGIAVEQDKN  776
KAPPA                  FTISVTTEILPVSMTKTSVDCTMYICGDSTECSNLLLQYGSFCTQLNRALTGIAVEQDKN  777
A 23.1                 FTISVTTEILPVSMTKTSVDCTMYICGDSTECSNLLLQYGSFCTQLNRALTGIAVEQDKN  777
IOTA                   FTISVTTEILPVSMTKTSVDCTMYICGDSTECSNLLLQYGSFCTQLNRALTGIAVEQDKN  777
THETA                  FTISVTTEILPVSMTKTSVDCTMYICGDSTECSNLLLQYGSFCTQLNRALTGIAVEQDKN  777
EPSILON                FTISVTTEILPVSMTKTSVDCTMYICGDSTECSNLLLQYGSFCTQLNRALTGIAVEQDKN  777
20 A.EU1               FTISVTTEILPVSMTKTSVDCTMYICGDSTECSNLLLQYGSFCTQLNRALTGIAVEQDKN  777
20 A.EU2               FTISVTTEILPVSMTKTSVDCTMYICGDSTECSNLLLQYGSFCTQLNRALTGIAVEQDKN  777
ZETA                   FTISVTTEILPVSMTKTSVDCTMYICGDSTECSNLLLQYGSFCTQLNRALTGIAVEQDKN  777
ALPHA                  FTISVTTEILPVSMTKTSVDCTMYICGDSTECSNLLLQYGSFCTQLNRALTGIAVEQDKN  774
ETA                    FTISVTTEILPVSMTKTSVDCTMYICGDSTECSNLLLQYGSFCTQLNRALTGIAVEQDKN  774
Omicron BA.1           FTISVTTEILPVSMTKTSVDCTMYICGDSTECSNLLLQYGSFCTQLKRALTGIAVEQDKN  774
Omicron BA.2_          FTISVTTEILPVSMTKTSVDCTMYICGDSTECSNLLLQYGSFCTQLKRALTGIAVEQDKN  774
Omicron BA.2.12.1      FTISVTTEILPVSMTKTSVDCTMYICGDSTECSNLLLQYGSFCTQLKRALTGIAVEQDKN  774
Omicron BA.4           FTISVTTEILPVSMTKTSVDCTMYICGDSTECSNLLLQYGSFCTQLKRALTGIAVEQDKN  772
Omicron BA.5           FTISVTTEILPVSMTKTSVDCTMYICGDSTECSNLLLQYGSFCTQLKRALTGIAVEQDKN  772
                       **********************************************:*************

wild type              TQEVFAQVKQIYKTPPIKDFGGFNFSQILPDPSKPSKRSFIEDLLFNKVTLADAGFIKQY  837
DELTA                  TQEVFAQVKQIYKTPPIKDFGGFNFSQILPDPSKPSKRSFIEDLLFNKVTLADAGFIKQY  835
LAMBDA                 TQEVFAQVKQIYKTPPIKDFGGFNFSQILPDPSKPSKRSFIEDLLFNKVTLADAGFIKQY  830
MU                     TQEVFAQVKQIYKTPPIKDFGGFNFSQILPDPSKPSKRSFIEDLLFNKVTLADAGFIKQY  838
BETA                   TQEVFAQVKQIYKTPPIKDFGGFNFSQILPDPSKPSKRSFIEDLLFNKVTLADAGFIKQY  834
GAMMA                  TQEVFAQVKQIYKTPPIKDFGGFNFSQILPDPSKPSKRSFIEDLLFNKVTLADAGFIKQY  837
B.1.1.318              TQEVFAQVKQIYKTPPIKHFGGFNFSQILPDPSKPSKRSFIEDLLFNKVTLADAGFIKQY  836
KAPPA                  TQEVFAQVKQIYKTPPIKDFGGFNFSQILPDPSKPSKRSFIEDLLFNKVTLADAGFIKQY  837
A 23.1                 TQEVFAQVKQIYKTPPIKDFGGFNFSQILPDPSKPSKRSFIEDLLFNKVTLADAGFIKQY  837
IOTA                   TQEVFAQVKQIYKTPPIKDFGGFNFSQILPDPSKPSKRSFIEDLLFNKVTLADAGFIKQY  837
THETA                  TQEVFAQVKQIYKTPPIKDFGGFNFSQILPDPSKPSKRSFIEDLLFNKVTLADAGFIKQY  837
EPSILON                TQEVFAQVKQIYKTPPIKDFGGFNFSQILPDPSKPSKRSFIEDLLFNKVTLADAGFIKQY  837
20 A.EU1               TQEVFAQVKQIYKTPPIKDFGGFNFSQILPDPSKPSKRSFIEDLLFNKVTLADAGFIKQY  837
20 A.EU2               TQEVFAQVKQIYKTPPIKDFGGFNFSQILPDPSKPSKRSFIEDLLFNKVTLADAGFIKQY  837
ZETA_                  TQEVFAQVKQIYKTPPIKDFGGFNFSQILPDPSKPSKRSFIEDLLFNKVTLADAGFIKQY  837
ALPHA_                 TQEVFAQVKQIYKTPPIKDFGGFNFSQILPDPSKPSKRSFIEDLLFNKVTLADAGFIKQY  834
ETA_                   TQEVFAQVKQIYKTPPIKDFGGFNFSQILPDPSKPSKRSFIEDLLFNKVTLADAGFIKQY  834
OmicromBA.1_           TQEVFAQVKQIYKTPPIKYFGGFNFSQILPDPSKPSKRSFIEDLLFNKVTLADAGFIKQY  834
OmicromBA.2_           TQEVFAQVKQIYKTPPIKYFGGFNFSQILPDPSKPSKRSFIEDLLFNKVTLADAGFIKQY  834
OmicromBA.2.12.1_      TQEVFAQVKQIYKTPPIKYFGGFNFSQILPDPSKPSKRSFIEDLLFNKVTLADAGFIKQY  834
OmicronBA.4_           TQEVFAQVKQIYKTPPIKYFGGFNFSQILPDPSKPSKRSFIEDLLFNKVTLADAGFIKQY  832
OmicronBA.5_           TQEVFAQVKQIYKTPPIKYFGGFNFSQILPDPSKPSKRSFIEDLLFNKVTLADAGFIKQY  832
                       ****************** *****************************************

wild type              GDCLGDIAARDLICAQKFNGLTVLPPLLTDEMIAQYTSALLAGTITSGWTFGAGAALQIP  897
DELTA                  GDCLGDIAARDLICAQKFNGLTVLPPLLTDEMIAQYTSALLAGTITSGWTFGAGAALQIP  895
LAMBDA                 GDCLGDIAARDLICAQKFNGLNVLPPLLTDEMIAQYTSALLAGTITSGWTFGAGAALQIP  890
MU                     GDCLGDIAARDLICAQKFNGLTVLPPLLTDEMIAQYTSALLAGTITSGWTFGAGAALQIP  898
BETA                   GDCLGDIAARDLICAQKFNGLTVLPPLLTDEMIAQYTSALLAGTITSGWTFGAGAALQIP  894
GAMMA                  GDCLGDIAARDLICAQKFNGLTVLPPLLTDEMIAQYTSALLAGTITSGWTFGAGAALQIP  897
B.1.1.318              GDCLGDIAARDLICAQKFNGLTVLPPLLTDEMIAQYTSALLAGTITSGWTFGAGAALQIP  896
KAPPA                  GDCLGDIAARDLICAQKFNGLTVLPPLLTDEMIAQYTSALLAGTITSGWTFGAGAALQIP  897
A 23.1                 GDCLGDIAARDLICAQKFNGLTVLPPLLTDEMIAQYTSALLAGTITSGWTFGAGAALQIP  897
IOTA                   GDCLGDIAARDLICAQKFNGLTVLPPLLTDEMIAQYTSALLAGTITSGWTFGAGAALQIP  897
THETA                  GDCLGDIAARDLICAQKFNGLTVLPPLLTDEMIAQYTSALLAGTITSGWTFGAGAALQIP  897
EPSILON                GDCLGDIAARDLICAQKFNGLTVLPPLLTDEMIAQYTSALLAGTITSGWTFGAGAALQIP  897
20 A.EU1               GDCLGDIAARDLICAQKFNGLTVLPPLLTDEMIAQYTSALLAGTITSGWTFGAGAALQIP  897
20 A.EU2               GDCLGDIAARDLICAQKFNGLTVLPPLLTDEMIAQYTSALLAGTITSGWTFGAGAALQIP  897
ZETA                   GDCLGDIAARDLICAQKFNGLTVLPPLLTDEMIAQYTSALLAGTITSGWTFGAGAALQIP  897
ALPHA                  GDCLGDIAARDLICAQKFNGLTVLPPLLTDEMIAQYTSALLAGTITSGWTFGAGAALQIP  894
ETA                    GDCLGDIAARDLICAQKFNGLTVLPPLLTDEMIAQYTSALLAGTITSGWTLGAGAALQIP  894
Omicron BA.1           GDCLGDIAARDLICAQKFKGLTVLPPLLTDEMIAQYTSALLAGTITSGWTFGAGAALQIP  894
Omicron BA.2           GDCLGDIAARDLICAQKFNGLTVLPPLLTDEMIAQYTSALLAGTITSGWTFGAGAALQIP  894
Omicron BA.2.12.1      GDCLGDIAARDLICAQKFNGLTVLPPLLTDEMIAQYTSALLAGTITSGWTFGAGAALQIP  894
Omicron BA.4           GDCLGDIAARDLICAQKFNGLTVLPPLLTDEMIAQYTSALLAGTITSGWTFGAGAALQIP  892
Omicron BA.5           GDCLGDIAARDLICAQKFNGLTVLPPLLTDEMIAQYTSALLAGTITSGWTFGAGAALQIP  892
                       ******************:**.****************************:*********
```

```
wild type           FAMQMAYRFNGIGVTQNVLYENQKLIANQFNSAIGKIQDSLSSTASALGKLQDVVNQNAQ  957
DELTA               FAMQMAYRFNGIGVTQNVLYENQKLIANQFNSAIGKIQDSLSSTASALGKLQNVVNQNAQ  955
LAMBDA              FAMQMAYRFNGIGVTQNVLYENQKLIANQFNSAIGKIQDSLSSTASALGKLQDVVNQNAQ  950
MU                  FAMQMAYRFNGIGVTQNVLYENQKLIANQFNSAIGKIQDSLSSTASALGKLQNVVNQNAQ  958
BETA                FAMQMAYRFNGIGVTQNVLYENQKLIANQFNSAIGKIQDSLSSTASALGKLQDVVNQNAQ  954
GAMMA               FAMQMAYRFNGIGVTQNVLYENQKLIANQFNSAIGKIQDSLSSTASALGKLQDVVNQNAQ  957
B.1.1.318           FAMQMAYRFNGIGVTQNVLYENQKLIANQFNSAIGKIQDSLSSTASALGKLQDVVNQNAQ  956
KAPPA               FAMQMAYRFNGIGVTQNVLYENQKLIANQFNSAIGKIQDSLSSTASALGKLQDVVNQNAQ  957
A 23.1              FAMQMAYRFNGIGVTQNVLYENQKLIANQFNSAIGKIQDSLSSTASALGKLQDVVNQNAQ  957
IOTA                FAMQMAYRFNGIGVTQNVLYENQKLIANQFNSAIGKIQDSLSSTASALGKLQDVVNQNAQ  957
THETA               FAMQMAYRFNGIGVTQNVLYENQKLIANQFNSAIGKIQDSLSSTASALGKLQDVVNQNAQ  957
EPSILON             FAMQMAYRFNGIGVTQNVLYENQKLIANQFNSAIGKIQDSLSSTASALGKLQDVVNQNAQ  957
20 A.EU1            FAMQMAYRFNGIGVTQNVLYENQKLIANQFNSAIGKIQDSLSSTASALGKLQDVVNQNAQ  957
20 A.EU2            FAMQMAYRFNGIGVTQNVLYENQKLIANQFNSAIGKIQDSLSSTASALGKLQDVVNQNAQ  957
ZETA                FAMQMAYRFNGIGVTQNVLYENQKLIANQFNSAIGKIQDSLSSTASALGKLQDVVNQNAQ  957
ALPHA               FAMQMAYRFNGIGVTQNVLYENQKLIANQFNSAIGKIQDSLSSTASALGKLQDVVNQNAQ  954
ETA                 FAMQMAYRFNGIGVTQNVLYENQKLIANQFNSAIGKIQDSLSSTASALGKLQDVVNQNAQ  954
Omicron BA.1        FAMQMAYRFNGIGVTQNVLYENQKLIANQFNSAIGKIQDSLSSTASALGKLQDVVNHNAQ  954
Omicron BA.2        FAMQMAYRFNGIGVTQNVLYENQKLIANQFNSAIGKIQDSLSSTASALGKLQDVVNHNAQ  954
Omicron BA.2.12.1   FAMQMAYRFNGIGVTQNVLYENQKLIANQFNSAIGKIQDSLSSTASALGKLQDVVNHNAQ  954
Omicron BA.4        FAMQMAYRFNGIGVTQNVLYENQKLIANQFNSAIGKIQDSLSSTASALGKLQDVVNHNAQ  952
Omicron BA.5        FAMQMAYRFNGIGVTQNVLYENQKLIANQFNSAIGKIQDSLSSTASALGKLQDVVNHNAQ  952
                    ****************************************************:***:***
```

```
wild type           ALNTLVKQLSSNFGAISSVLNDILSRLDKVEAEVQIDRLITGRLQSLQTYVTQQLIRAAE  1017
DELTA               ALNTLVKQLSSNFGAISSVLNDILSRLDKVEAEVQIDRLITGRLQSLQTYVTQQLIRAAE  1015
LAMBDA              ALNTLVKQLSSNFGAISSVLNDILSRLDKVEAEVQIDRLITGRLQSLQTYVTQQLIRAAE  1010
MU                  ALNTLVKQLSSNFGAISSVLNDILSRLDKVEAEVQIDRLITGRLQSLQTYVTQQLIRAAE  1018
BETA                ALNTLVKQLSSNFGAISSVLNDILSRLDKVEAEVQIDRLITGRLQSLQTYVTQQLIRAAE  1014
GAMMA               ALNTLVKQLSSNFGAISSVLNDILSRLDKVEAEVQIDRLITGRLQSLQTYVTQQLIRAAE  1017
B.1.1.318           ALNTLVKQLSSNFGAISSVLNDILSRLDKVEAEVQIDRLITGRLQSLQTYVTQQLIRAAE  1016
KAPPA               ALNTLVKQLSSNFGAISSVLNDILSRLDKVEAEVQIDRLITGRLQSLQTYVTQQLIRAAE  1017
A 23.1              ALNTLVKQLSSNFGAISSVLNDILSRLDKVEAEVQIDRLITGRLQSLQTYVTQQLIRAAE  1017
IOTA                ALNTLVKQLSSNFGAISSVLNDILSRLDKVEAEVQIDRLITGRLQSLQTYVTQQLIRAAE  1017
THETA               ALNTLVKQLSSNFGAISSVLNDILSRLDKVEAEVQIDRLITGRLQSLQTYVTQQLIRAAE  1017
EPSILON             ALNTLVKQLSSNFGAISSVLNDILSRLDKVEAEVQIDRLITGRLQSLQTYVTQQLIRAAE  1017
20 A.EU1            ALNTLVKQLSSNFGAISSVLNDILSRLDKVEAEVQIDRLITGRLQSLQTYVTQQLIRAAE  1017
20 A.EU2            ALNTLVKQLSSNFGAISSVLNDILSRLDKVEAEVQIDRLITGRLQSLQTYVTQQLIRAAE  1017
ZETA                ALNTLVKQLSSNFGAISSVLNDILSRLDKVEAEVQIDRLITGRLQSLQTYVTQQLIRAAE  1017
ALPHA               ALNTLVKQLSSNFGAISSVLNDILARLDKVEAEVQIDRLITGRLQSLQTYVTQQLIRAAE  1014
ETA                 ALNTLVKQLSSNFGAISSVLNDILSRLDKVEAEVQIDRLITGRLQSLQTYVTQQLIRAAE  1014
Omicron BA.1        ALNTLVKQLSSKFGAISSVLNDIFSRLDKVEAEVQIDRLITGRLQSLQTYVTQQLIRAAE  1014
Omicron BA.2        ALNTLVKQLSSKFGAISSVLNDILSRLDKVEAEVQIDRLITGRLQSLQTYVTQQLIRAAE  1014
Omicron BA.2.12.1   ALNTLVKQLSSKFGAISSVLNDILSRLDKVEAEVQIDRLITGRLQSLQTYVTQQLIRAAE  1014
Omicron BA.4        ALNTLVKQLSSKFGAISSVLNDILSRLDKVEAEVQIDRLITGRLQSLQTYVTQQLIRAAE  1012
Omicron BA.5        ALNTLVKQLSSKFGAISSVLNDILSRLDKVEAEVQIDRLITGRLQSLQTYVTQQLIRAAE  1012
                    ***********:***********::***********************************
```

```
wild type           IRASANLAATKMSECVLGQSKRVDFCGKGYHLMSFPQSAPHGVVFLHVTYVPAQEKNFTT  1077
DELTA               IRASANLAATKMSECVLGQSKRVDFCGKGYHLMSFPQSAPHGVVFLHVTYVPAQEKNFTT  1075
LAMBDA              IRASANLAATKMSECVLGQSKRVDFCGKGYHLMSFPQSAPHGVVFLHVTYVPAQEKNFTT  1070
MU                  IRASANLAATKMSECVLGQSKRVDFCGKGYHLMSFPQSAPHGVVFLHVTYVPAQEKNFTT  1078
BETA                IRASANLAATKMSECVLGQSKRVDFCGKGYHLMSFPQSAPHGVVFLHVTYVPAQEKNFTT  1074
GAMMA               IRASANLAAIKMSECVLGQSKRVDFCGKGYHLMSFPQSAPHGVVFLHVTYVPAQEKNFTT  1077
B.1.1.318           IRASANLAATKMSECVLGQSKRVDFCGKGYHLMSFPQSAPHGVVFLHVTYVPAQEKNFTT  1076
KAPPA               IRASANLAATKMSECVLGQSKRVDFCGKGYHLMSFPQSAPHGVVFLHVTYVPAHEKNFTT  1077
A 23.1              IRASANLAATKMSECVLGQSKRVDFCGKGYHLMSFPQSAPHGVVFLHVTYVPAQEKNFTT  1077
IOTA                IRASANLAATKMSECVLGQSKRVDFCGKGYHLMSFPQSAPHGVVFLHVTYVPAQEKNFTT  1077
THETA               IRASANLAATKMSECVLGQSKRVDFCGKGYHLMSFPQSAPHGVVFLHVTYVPAQEKNFTT  1077
EPSILON             IRASANLAATKMSECVLGQSKRVDFCGKGYHLMSFPQSAPHGVVFLHVTYVPAQEKNFTT  1077
20 A.EU1            IRASANLAATKMSECVLGQSKRVDFCGKGYHLMSFPQSAPHGVVFLHVTYVPAQEKNFTT  1077
20 A.EU2            IRASANLAATKMSECVLGQSKRVDFCGKGYHLMSFPQSAPHGVVFLHVTYVPAQEKNFTT  1077
ZETA                IRASANLAATKMSECVLGQSKRVDFCGKGYHLMSFPQSAPHGVVFLHVTYVPAQEKNFTT  1077
ALPHA               IRASANLAATKMSECVLGQSKRVDFCGKGYHLMSFPQSAPHGVVFLHVTYVPAQEKNFTT  1074
ETA                 IRASANLAATKMSECVLGQSKRVDFCGKGYHLMSFPQSAPHGVVFLHVTYVPAQEKNFTT  1074
Omicron BA.1        IRASANLAATKMSECVLGQSKRVDFCGKGYHLMSFPQSAPHGVVFLHVTYVPAQEKNFTT  1074
Omicron BA.2        IRASANLAATKMSECVLGQSKRVDFCGKGYHLMSFPQSAPHGVVFLHVTYVPAQEKNFTT  1074
Omicron BA.2.12.1   IRASANLAATKMSECVLGQSKRVDFCGKGYHLMSFPQSAPHGVVFLHVTYVPAQEKNFTT  1074
Omicron BA.4        IRASANLAATKMSECVLGQSKRVDFCGKGYHLMSFPQSAPHGVVFLHVTYVPAQEKNFTT  1072
Omicron BA.5        IRASANLAATKMSECVLGQSKRVDFCGKGYHLMSFPQSAPHGVVFLHVTYVPAQEKNFTT  1072
                    ********* *******************************************:******
```

```
wild type            APAICHDGKAHFPREGVFVSNGTHWFVTQRNFYEPQIITTDNTFVSGNCDVVIGIVNNTV  1137
DELTA                APAICHDGKAHFPREGVFVSNGTHWFVTQRNFYEPQIITTDNTFVSGNCDVVIGIVNNTV  1135
LAMBDA               APAICHDGKAHFPREGVFVSNGTHWFVTQRNFYEPQIITTDNTFVSGNCDVVIGIVNNTV  1130
MU                   APAICHDGKAHFPREGVFVSNGTHWFVTQRNFYEPQIITTDNTFVSGNCDVVIGIVNNTV  1138
BETA                 APAICHDGKAHFPREGVFVSNGTHWFVTQRNFYEPQIITTDNTFVSGNCDVVIGIVNNTV  1134
GAMMA                APAICHDGKAHFPREGVFVSNGTHWFVTQRNFYEPQIITTDNTFVSGNCDVVIGIVNNTV  1137
B.1.1.318            APAICHDGKAHFPREGVFVSNGTHWFVTQRNFYEPQIITTDNTFVSGNCDVVIGIVNNTV  1136
KAPPA                APAICHDGKAHFPREGVFVSNGTDWFVTQRNFYEPQIITTDNTFVSGNCDVVIGIVNNTV  1137
A 23.1               APAICHDGKAHFPREGVFVSNGTHWFVTQRNFYEPQIITTDNTFVSGNCDVVIGIVNNTV  1137
IOTA                 APAICHDGKAHFPREGVFVSNGTHWFVTQRNFYEPQIITTDNTFVSGNCDVVIGIVNNTV  1137
THETA                APAICHDGKAHFPRKGVFVSNGTYWFVTQRNFYEPQIITTDNTFVSGNCDVVIGIVNNTV  1137
EPSILON              APAICHDGKAHFPREGVFVSNGTHWFVTQRNFYEPQIITTDNTFVSGNCDVVIGIVNNTV  1137
20 A.EU1             APAICHDGKAHFPREGVFVSNGTHWFVTQRNFYEPQIITTDNTFVSGNCDVVIGIVNNTV  1137
20 A.EU2             APAICHDGKAHFPREGVFVSNGTHWFVTQRNFYEPQIITTDNTFVSGNCDVVIGIVNNTV  1137
ZETA                 APAICHDGKAHFPREGVFVSNGTHWFVTQRNFYEPQIITTDNTFVSGNCDVVIGIVNNTV  1137
ALPHA                APAICHDGKAHFPREGVFVSNGTHWFVTQRNFYEPQIITTHNTFVSGNCDVVIGIVNNTV  1134
ETA                  APAICHDGKAHFPREGVFVSNGTHWFVTQRNFYEPQIITTDNTFVSGNCDVVIGIVNNTV  1134
Omicron BA.1         APAICHDGKAHFPREGVFVSNGTHWFVTQRNFYEPQIITTDNTFVSGNCDVVIGIVNNTV  1134
Omicron BA.2         APAICHDGKAHFPREGVFVSNGTHWFVTQRNFYEPQIITTDNTFVSGNCDVVIGIVNNTV  1134
Omicron BA.2.12.1    APAICHDGKAHFPREGVFVSNGTHWFVTQRNFYEPQIITTDNTFVSGNCDVVIGIVNNTV  1134
Omicron BA.4         APAICHDGKAHFPREGVFVSNGTHWFVTQRNFYEPQIITTDNTFVSGNCDVVIGIVNNTV  1132
Omicron BA.5         APAICHDGKAHFPREGVFVSNGTHWFVTQRNFYEPQIITTDNTFVSGNCDVVIGIVNNTV  1132
                     **************:******** ****************.*******************

wild type            YDPLQPELDSFKEELDKYFKNHTSPDVDLGDISGINASVVNIQKEIDRLNEVAKNLNESL  1197
DELTA                YDPLQPELDSFKEELDKYFKNHTSPDVDLGDISGINASVVNIQKEIDRLNEVAKNLNESL  1195
LAMBDA               YDPLQPELDSFKEELDKYFKNHTSPDVDLGDISGINASVVNIQKEIDRLNEVAKNLNESL  1190
MU                   YDPLQPELDSFKEELDKYFKNHTSPDVDLGDISGINASVVNIQKEIDRLNEVAKNLNESL  1198
BETA                 YDPLQPELDSFKEELDKYFKNHTSPDVDLGDISGINASVVNIQKEIDRLNEVAKNLNESL  1194
GAMMA                YDPLQPELDSFKEELDKYFKNHTSPDVDLGDISGINASFVNIQKEIDRLNEVAKNLNESL  1197
B.1.1.318            YDPLQPELDSFKEELDKYFKNHTSPDVDLGDISGINASVVNIQKEIDRLNEVAKNLNESL  1196
KAPPA                YDPLQPELDSFKEELDKYFKNHTSPDVDLGDISGINASVVNIQKEIDRLNEVAKNLNESL  1197
A 23.1               YDPLQPELDSFKEELDKYFKNHTSPDVDLGDISGINASVVNIQKEIDRLNEVAKNLNESL  1197
IOTA                 YDPLQPELDSFKEELDKYFKNHTSPDVDLGDISGINASVVNIQKEIDRLNEVAKNLNESL  1197
THETA                YDPLQPELDSFKEELDKYFKNHTSPDVDLGDISGINASFVNIQKEIDRLNEVAKNLNESL  1197
EPSILON              YDPLQPELDSFKEELDKYFKNHTSPDVDLGDISGINASVVNIQKEIDRLNEVAKNLNESL  1197
20 A.EU1             YDPLQPELDSFKEELDKYFKNHTSPDVDLGDISGINASVVNIQKEIDRLNEVAKNLNESL  1197
20 A.EU2             YDPLQPELDSFKEELDKYFKNHTSPDVDLGDISGINASVVNIQKEIDRLNEVAKNLNESL  1197
ZETA                 YDPLQPELDSFKEELDKYFKNHTSPDVDLGDISGINASFVNIQKEIDRLNEVAKNLNESL  1197
ALPHA                YDPLQPELDSFKEELDKYFKNHTSPDVDLGDISGINASVVNIQKEIDRLNEVAKNLNESL  1194
ETA                  YDPLQPELDSFKEELDKYFKNHTSPDVDLGDISGINASVVNIQKEIDRLNEVAKNLNESL  1194
Omicron BA.1         YDPLQPELDSFKEELDKYFKNHTSPDVDLGDISGINASVVNIQKEIDRLNEVAKNLNESL  1194
Omicron BA.2         YDPLQPELDSFKEELDKYFKNHTSPDVDLGDISGINASVVNIQKEIDRLNEVAKNLNESL  1194
Omicron BA.2.12.1    YDPLQPELDSFKEELDKYFKNHTSPDVDLGDISGINASVVNIQKEIDRLNEVAKNLNESL  1194
Omicron BA.4         YDPLQPELDSFKEELDKYFKNHTSPDVDLGDISGINASVVNIQKEIDRLNEVAKNLNESL  1192
Omicron BA.5         YDPLQPELDSFKEELDKYFKNHTSPDVDLGDISGINASVVNIQKEIDRLNEVAKNLNESL  1192
                     **************************************.*********************

wild type            IDLQELGKYEQYIKWPWYIWLGFIAGLIAIVMVTIMLCCMTSCCSCLKGCCSCGSCCKFD  1257
DELTA                IDLQELGKYEQYIKWPWYIWLGFIAGLIAIVMVTIMLCCMTSCCSCLKGCCSCGSCCKFD  1255
LAMBDA               IDLQELGKYEQYIKWPWYIWLGFIAGLIAIVMVTIMLCCMTSCCSCLKGCCSCGSCCKFD  1250
MU                   IDLQELGKYEQYIKWPWYIWLGFIAGLIAIVMVTIMLCCMTSCCSCLKGCCSCGSCCKFD  1258
BETA                 IDLQELGKYEQYIKWPWYIWLGFIAGLIAIVMVTIMLCCMTSCCSCLKGCCSCGSCCKFD  1254
GAMMA                IDLQELGKYEQYIKWPWYIWLGFIAGLIAIVMVTIMLCCMTSCCSCLKGCCSCGSCCKFD  1257
B.1.1.318            IDLQELGKYEQYIKWPWYIWLGFIAGLIAIVMVTIMLCCMTSCCSCLKGCCSCGSCCKFD  1256
KAPPA                IDLQELGKYEQYIKWPWYIWLGFIAGLIAIVMVTIMLCCMTSCCSCLKGCCSCGSCCKFD  1257
A 23.1               IDLQELGKYEQYIKWPWYIWLGFIAGLIAIVMVTIMLCCMTSCCSCLKGCCSCGSCCKFD  1257
IOTA                 IDLQELGKYEQYIKWPWYIWLGFIAGLIAIVMVTIMLCCMTSCCSCLKGCCSCGSCCKFD  1257
THETA                IDLQELGKYEQYIKWPWYIWLGFIAGLIAIVMVTIMLCCMTSCCSCLKGCCSCGSCCKFD  1257
EPSILON              IDLQELGKYEQYIKWPWYIWLGFIAGLIAIVMVTIMLCCMTSCCSCLKGCCSCGSCCKFD  1257
20 A.EU1             IDLQELGKYEQYIKWPWYIWLGFIAGLIAIVMVTIMLCCMTSCCSCLKGCCSCGSCCKFD  1257
20 A.EU2             IDLQELGKYEQYIKWPWYIWLGFIAGLIAIVMVTIMLCCMTSCCSCLKGCCSCGSCCKFD  1257
ZETA                 IDLQELGKYEQYIKWPWYIWLGFIAGLIAIVMVTIMLCCMTSCCSCLKGCCSCGSCCKFD  1257
ALPHA                IDLQELGKYEQYIKWPWYIWLGFIAGLIAIVMVTIMLCCMTSCCSCLKGCCSCGSCCKFD  1254
ETA                  IDLQELGKYEQYIKWPWYIWLGFIAGLIAIVMVTIMLCCMTSCCSCLKGCCSCGSCCKFD  1254
Omicron BA.1         IDLQELGKYEQYIKWPWYIWLGFIAGLIAIVMVTIMLCCMTSCCSCLKGCCSCGSCCKFD  1254
Omicron BA.2         IDLQELGKYEQYIKWPWYIWLGFIAGLIAIVMVTIMLCCMTSCCSCLKGCCSCGSCCKFD  1254
Omicron BA.2.12.1    IDLQELGKYEQYIKWPWYIWLGFIAGLIAIVMVTIMLCCMTSCCSCLKGCCSCGSCCKFD  1254
Omicron BA.4         IDLQELGKYEQYIKWPWYIWLGFIAGLIAIVMVTIMLCCMTSCCSCLKGCCSCGSCCKFD  1252
Omicron BA.5         IDLQELGKYEQYIKWPWYIWLGFIAGLIAIVMVTIMLCCMTSCCSCLKGCCSCGSCCKFD  1252
                     ************************************************************
```

```
wild type             EDDSEPVLKGVKLHYT   1273
DELTA                 EDDSEPVLKGVKLHYT   1271
LAMBDA                EDDSEPVLKGVKLHYT   1266
MU                    EDDSEPVLKGVKLHYT   1274
BETA                  EDDSEPVLKGVKLHYT   1270
GAMMA                 EDDSEPVLKGVKLHYT   1273
B.1.1.318             EDDSEPVLKGVKLHYT   1272
KAPPA                 EDDSEPVLKGVKLHYT   1273
A 23.1                EDDSEPVLKGVKLHYT   1273
IOTA                  EDDSEPVLKGVKLHYT   1273
THETA                 EDDSEPVLKGVKLHYT   1273
EPSILON               EDDSEPVLKGVKLHYT   1273
20 A.EU1              EDDSEPVLKGVKLHYT   1273
20 A.EU2              EDDSEPVLKGVKLHYT   1273
ZETA                  EDDSEPVLKGVKLHYT   1273
ALPHA                 EDDSEPVLKGVKLHYT   1270
ETA                   EDDSEPVLKGVKLHYT   1270
Omicron BA.1          EDDSEPVLKGVKLHYT   1270
Omicron BA.2          EDDSEPVLKGVKLHYT   1270
Omicron BA.2.12.1     EDDSEPVLKGVKLHYT   1270
Omicron BA.4          EDDSEPVLKGVKLHYT   1268
Omicron BA.5          EDDSEPVLKGVKLHYT   1268
                      ****************
```

Figure S1. Sequences alignment. SARS-CoV-2 wild type spike protein and its most diffused variants were aligned by using the Clustal Omega program (https://www.ebi.ac.uk/Tools/msa/clustalo/). The parameters used were used by default of the program. In yellow are underlined wild type residues with high rate of mutation. In red are underlined mutations present only in a single variant. In green are underlined mutations present only in all the Omicron variants. In cyan are underlined mutations present in all the Omicron variants excluding Omicron BA.1.
